# Proteomics Characterization of Cytoplasmic and Lipid-Associated Membrane Proteins of Human Pathogen *Mycoplasma fermentans* M64

**DOI:** 10.1371/journal.pone.0035304

**Published:** 2012-04-20

**Authors:** Yi-Chang Liu, I-Hsuan Lin, Wei-Jen Chung, Wensi S. Hu, Wailap Victor Ng, Chi-Yu Lu, Tsung-Yen Huang, Hung-Wei Shu, Kwang-Jen Hsiao, Shih-Feng Tsai, Chuan-Hsiung Chang, Chao-Hsiung Lin

**Affiliations:** 1 Institute of Biopharmaceutical Research, National Yang-Ming University, Taipei, Taiwan; 2 Institute of Biomedical Informatics, National Yang-Ming University, Taipei, Taiwan; 3 Bioinformatics Program, Taiwan International Graduate Program, Academia Sinica, Taipei, Taiwan; 4 Institute of Biotechnology in Medicine, National Yang-Ming University, Taipei, Taiwan; 5 Proteomics Research Center, National Yang-Ming University, Taipei, Taiwan; 6 Department of Life Sciences and Institute of Genome Sciences, National Yang-Ming University, Taipei, Taiwan; 7 Department of Medical Research and Education, Taipei Veterans General Hospital, Taipei, Taiwan; 8 Division of Molecular and Genomic Medicine, National Health Research Institutes, Zhunan, Taiwan; I2MC INSERM UMR U1048, France

## Abstract

*Mycoplasma fermentans* is a potent human pathogen which has been implicated in several diseases. Notably, its lipid-associated membrane proteins (LAMPs) play a role in immunomodulation and development of infection-associated inflammatory diseases. However, the systematic protein identification of pathogenic *M. fermentans* has not been reported. From our recent sequencing results of *M. fermentans* M64 isolated from human respiratory tract, its genome is around 1.1 Mb and encodes 1050 predicted protein-coding genes. In the present study, soluble proteome of *M. fermentans* was resolved and analyzed using two-dimensional gel electrophoresis. In addition, Triton X-114 extraction was carried out to enrich amphiphilic proteins including putative lipoproteins and membrane proteins. Subsequent mass spectrometric analyses of these proteins had identified a total of 181 *M. fermentans* ORFs. Further bioinformatics analysis of these ORFs encoding proteins with known or so far unknown orthologues among bacteria revealed that a total of 131 proteins are homologous to known proteins, 11 proteins are conserved hypothetical proteins, and the remaining 39 proteins are likely *M. fermentans*-specific proteins. Moreover, Triton X-114-enriched fraction was shown to activate NF-kB activity of raw264.7 macrophage and a total of 21 lipoproteins with predicted signal peptide were identified therefrom. Together, our work provides the first proteome reference map of *M. fermentans* as well as several putative virulence-associated proteins as diagnostic markers or vaccine candidates for further functional study of this human pathogen.

## Introduction

Mycoplasmas belong to the Mollicutes class of organisms and are often considered as wall-less bacteria with the smallest genomes. Colonization of Mollicutes has been found in both plants and animals, where many animal mycoplasmas are extracellular parasites and plant mycoplasmas are localized solely in the phloem sieve tubes of affected plants and transmitted by insect vectors [1], [2]. Scientists have isolated at least 16 species of Mycoplasma from humans and their major colonization sites include oropharynx, upper respiratory tract, and genitourinary tract [1]. Table S1 summarized a list of several Mollicutes species and their hosts. *Mycoplasma fermentans*, a human cell-invasive mycoplasma, has been suspected to associate with several human diseases. For example, presence of *M. fermentans* among acquired immunodeficiency syndrome (AIDS) patients was reported in 1989 [3]. *M. fermentans* and other two mycoplasmas were proposed as cofactors of human immunodeficiency virus (HIV) for transmission and progression of virulence [4], [5]. In addition, *M. fermentans* was also linked to the prevalence of rheumatoid arthritis [6]. The potential of *M. fermentans* to interact with the immune system has been intensively investigated and molecular basis of *M. fermentans* as an immunomodulatory agent has been reviewed [7], [8]. Moreover, studies have shown that lipid-associated membrane proteins (LAMPs) of *M. fermentans* can interact with monocytes and macrophages [9]. It has been shown that the LAMPs of *M. fermentans* and *M. penetrans* can activate the long-terminal repeats of human immunodeficiency virus through Toll-like receptors (TLRs) [5]. Accordingly, the interaction of *M. fermentans*-derived LAMPs with cells of the innate immune system could be via TLRs, much like the process found in *M. genitalium*, and ultimately lead to the NF-kB activation, release of proinflammatory cytokines and induction of apoptotic cell death [10], [11].

Systematic study of the constitutive proteome in pathogens can certainly provide valuable information about how they regulate metabolism and pathogenesis [12], [13]. In this study, we performed the proteome analysis of *M. fermentans* M64, a local strain isolated from human respiratory tract [14] and whose genome has been recently sequenced [15]. Protein expression profiles of this microorganism were established using two-dimensional gel electrophoresis (2-DE) and further characterized for protein identities using mass spectrometry-based peptide mass fingerprint (PMF) or amino acid sequencing. Subsequent bioinformatics annotation of the identified proteins reveals the functional categorization as well as the comparison to other Mycoplasmas. Several pathways of *M. fermentans* in the host-free environment are proposed. In addition, a number of LAMPs identified from the NF-kB activating fraction of *M. fermentans* proteome were suggested for further functional study.

## Materials and Methods

### Materials

Tris, acetone, bromophenol blue, Coomassie® Brilliant Blue G-250, methanol, acetic acid, ACN, and TFA were purchased from Merck KGaA (Darmstadt, Germany). Urea, thiourea, DTT, triphenylphospine (TPP), iodoacetamide, ammonium persulfate, SDS, glycine, glycerol, silver nitrate, and CHCA were purchased from Acros Organics (Geel, Gelgium). Ammonium sulfate, potassium chloride, sodium thiosulfate, phosphoric acid, and sodium carbonate were purchased from Ferax (Berlin, Germany). Protein Assay Rapid Kit and ammonium bicarbonate were purchased from Wako Pure Chemical Industries Ltd. (Osaka, Japan). Triton X-100, acrylamide, methylenebisacrylamide, TEMED, Immoboline IPG Drystrips, IPG buffers, and Precision Plus protein standards were purchased from Amersham Biosciences (Uppsala, Sweden). C@mplete™ Mini protease inhibitor was purchased from Roche (Basel, Switzerland). Modified trypsin was purchased from Promega (Madison, USA). Triton X-114 and benzonase were purchased from Sigma (St. Louis, USA). EZ-Link Sulfo-NHS-LC-Biotin and NeutrAvidin were purchased from Pierce Biotechnology (Rockford, USA). All reagents for luciferase assays were purchased from Promega (Madison, WI).

### Apparatus

Immoboline Drystrip Reswelling Trays, Ettan IPGphor platform, Ettan DALTsix Large Vertical System, SG 500 Gradient Maker, MultiTemp III Termostatic Circulator 115 VAC, EPS Power Supply, and Typhoon 9400 imager were purchased from Amersham Biosciences (Uppsala, Sweden). ProteomeWorks Spot Cuter and 2-D image analysis software PDQuest were purchased from Bio-Rad Laboratories Inc. (Hercules, USA). AnchorChip, Ultraflex II MALDI-TOF/TOF mass spectrometer, FlexAnalysis software v2.2 and Biotool software were purchased from Bruker Daltonics (Breman, Germany). C18 reversed-phase column was purchased from Microtech Scientific (Vista, CA, USA). CapLC system 1100 series was purchased from Agilent Technologies (Palo Alto, CA, USA). The quadrupole time-of-flight mass spectrometer (QTOF2) and Masslynx v4.0 was purchased from Micromass (Manchester, UK).

### Culture and protein extraction of *M. fermentans* M64

*M. fermentans* M64 strain was grown anaerobically in 400 ml of SP-4 medium at 37°C overnight [14]. Cells were collected by centrifugation at 13,000 g at 4°C for 30 min. The cell pellet was washed by PBS followed by centrifugation at 13,000 g at 4°C for 30 min. The resulting cell pellet was resuspended in 40 mM Tris buffer containing 7 M urea, 2 M thiourea, 4% Triton ×100, and 250 units of benzonase for violent vertex for 2 min, followed by incubation in room temperature for 30 min. The insoluble cell debris was collected by centrifugation at 13,000 g at 4°C for 30 min, and was then discarded. To remove water-soluble contaminants such as salt, four times of the sample volume of cold acetone was added, and the solution was incubated at −20°C overnight. Precipitated proteins were collected by centrifugation at 13,000 g for 30 min. Proteins were dissolved in the same buffer without benzonase, and the concentration was determined by Wako Protein Assay Rapid Kit. Samples were stored in aliquots at −80°C.

### 2-DE

2-DE was performed according to the renewed protocol by Görg and colleagues in 2000 [16], with some modifications. For rehydration loading, samples were mixed with 7 M urea, 2 M thiourea, 4% Triton ×100, 0.4% IPG buffer, 100 mM DTT, 2 mM TPP, and 0.002% w/v bromophenol blue. IPG strips were rehydrated with this sample solution for 12 hours in the rehydration tray. Isoelectric focusing was then performed with voltage being gradually increased to 8,000 V, and focused for 40000∼64000 Voltage-hour. For cup-loading, IPG strip was first rehydrated by the rehydration solution described above without protein samples for six hours, and 100 µl protein sample was then added to the cup placed near the anode side for a similar isoelectric focusing condition as described above. To prepare SDS-PAGE separation, focused IPG strips were equilibrated in the equilibrium buffer containing 6 M urea, 50 mM Tris-HCl pH 8.8, 15% glycerol, 2% w/v SDS, and 1% w/v DTT for 15 min, followed by the second equilibration with the same buffer except that DTT was replaced by 2.5% w/v iodoacetamide. IPG strips were then placed onto SDS polyacrylamide gels, and sealed by 0.5% w/v agarose with 0.002% bromophenol blue. SDS-PAGE was performed until the dye front had reached the bottom of gels. Gels were fixed in 40% methanol and 10% acetic acid for 30 min and were then stained by Coomassie® blue or sliver. For Coomassie® blue staining, fixed gels were first washed by deionized water for 15 min and were then incubated in the staining solution containing 17% w/v ammonium sulfate, 3% phosphoric acid, 34% methanol, and 0.1% w/v Coomassie® blue G-250 overnight. The silver staining procedure was according to Blum et al. for convenience of further MS analysis [17]. Briefly, fixed gels were incubated in 30% methanol for 15 min, washed by Milli-Q water several times, and incubated in 0.05% w/v sodium thiosulfate for 2 min. Gels were then washed for 30 seconds several times, and then incubated in 0.2% silver nitrate for 25 min. Gels were washed shortly and developed with 3% w/v sodium carbonate, 0.001% w/v sodium thiosulfate and 0.02% formaldehyde until the desired spot profiles were obtained. Development was stopped by 1.4% EDTA. Gels were then scanned by Microtek ScanMaker 8700. Images were analyzed by PDQuest image analyzing software.

### In-gel digestion

Protein spots were cut from gels into 1∼2 mm^3^ pieces. Coomassie® blue G-250 stained gel pieces were then destained by 50% ACN and 25 mM ammonium bicarbonate. Silver stained gel pieces were destained by 15 mM potassium ferricyanide and 50 mM sodium thiosulfate [18]. Destained gel pieces were then washed with 50 mM ammonium bicarbonate followed by 100% ACN. Wash was repeated by two or three times. Gel pieces were dehydrated by ACN followed by SpeedVac drying. For a single piece of gel, 40 ng of trypsin dissolved in 25 mM ammonium bicarbonate was applied onto the gel and incubated at 4°C for 30 min. Additional 25 mM ammonium bicarbonate was then applied to the pieces to prevent dehydration during digestion. Digestion was performed for 1 hour at 55°C [19]. Extraction was then performed by applying 75% acetonitrile and 1% TFA onto gels with 10 min sonication for three to four times. The solution extracted was then concentrated by SpeedVac drying.

### MS Analysis

For PMF analysis by MALDI-TOF MS, 0.5 µl sample solution was mixed with 0.5 µl 2 mg/ml CHCA matrix solution and applied onto the AnchorChip 600 target plate. MALDI analysis was performed on the Ultraflex II mass spectrometer with a 337 nm wavelength nitrogen laser. All samples were analyzed in a positive reflectron mode. An average of approximately 400 shots was acquired for each spot. Calibration was done externally by 6 single charged peptides, including angiotensin II ([M+H]^+^ = 1046.5418), angiotensin I ([M+H]^+^ = 1296.6848), substance P ([M+H]^+^ = 1347.7354), bombesin ([M+H]^+^ = 1619.8223), ACTH clip 1–17 ([M+H]^+^ = 2093.09), and ACTH clip 18–39 ([M+H]^+^ = 2465.1983). Spectra generated were then analyzed by FlexAnalysis software version 2.2.

For sequencing analysis by LC-MS/MS, the tryptic peptides were loaded and separated in a 150×10 mm C18 reversed-phase column using CapLC system with a gradient solution of 5 to 75% ACN aqueous solution over 45 min. Peptides were eluted at 200 nl/min directly into spray source of QTOF2. The QTOF2 is fitted with an electrospray in an orthogonal configuration with a Z-SPRAY interface. The source block temperature was set to 80°C and cone voltage was kept at 25 V. The quadrupole analyzer was used to select precursor ions for subsequent fragmentation in the hexapole collision cell. The argon was used as collision gas and the collision voltage was maintained at 20–30 V to give optimal fragmentation. The fragmented ion products were analyzed in an orthogonal time-of-flight analyzer fitted with a reflector, a micro-channel plate detector and a time-to-digital converter. The mass spectrum acquisitions were recorded within the mass range of 400–2000 m/z and the tandem mass spectra were recorded within 50–3500 m/z. The recorded spectral data were processed with MassLynx, version 4.0 to give centroid MS/MS data.

### In-house MASCOT database construction and ORF match

All protein spots were excised, trypsin-digested, and subjected to a MALDI-TOF MS for PMF using an in-house MASCOT software v2.2 (Matrix Science, London, UK). A database of 1050 *M. fermentans* M64 ORFs was established in local server for protein identification. If the MALDI spectra failed to provide confident match, the amino acid sequencing using ESI-MS/MS is further carried out to obtain protein identities. For PMF analysis, peak lists generated from FlexAnalysis were then sent to Biotool software for identity searching on the in-house MASCOT server. The charge state was set to [M+H]^+^ with tolerance of ±200 ppm. The default tryptic cleavage rule of the MASCOT server was used, and only one missed cut is allowed. Protein matches with expectation values less than 0.05 according to MASCOT assignment were accepted. For sequencing analysis, the pkl-format spectra of QTOF2 were directly searched against in-house MASCOT database. The charge state was set at [M+2H]^2+^ and [M+3H]^3+^ with peptide tolerance of ±1.2 Da and MS/MS tolerance of ±0.6 Da. Again, the default tryptic cleavage rule of the MASCOT server was used, and only one missed cut is allowed. Peptide matches with expectation values less than 0.01 were accepted.

### Isolation of lipoproteins by Triton X-114 phase separation

Cells were treated with Triton X-114 to extract membrane-associated proteins according to the method as previously described [20], [21], with some modifications. Cell pellet collected from the centrifugation of 400-ml culture medium described in section 2.3 was suspended in 0.9 ml of TS buffer (50 mM pH 7.6 Tris-HCl, 150 mM sodium chloride, and C@mplete™ Mini protease inhibitor). The cell suspension was added with 0.1 ml of 20% Triton X-114 solution and shaken gently at 4°C for 2 hr. The insoluble materials were removed by centrifugation at 10,000 g for 10 min at 4°C. The supernatant was transferred to a new eppendorf and incubated at 37°C for 5 min for phase separation followed by centrifugation at 10,000 g for 5 min. The upper aqueous phase was discarded, and 0.9 ml of TS buffer was added. The solution was treated again as described above. Lipoproteins extracted from Triton X-114 phase were then precipitated by adding nine times the volumes of methanol. The precipitated proteins were resuspended in 0.1% SDS solution for measuring concentration with Bradford reagent (Sigma). The 20 µg of proteins were then diluted with sample buffer (62.5 mM Tris-Hcl (pH = 6.8), 2% w/v SDS, 10% glycerol, 0.01% bromphenol blue) and separated by 10% SDS-PAGE with Coomassie Brilliant Blue G-250 staining. In-gel digestion and MS analysis were then carried out as described above.

### Measurement of NF-kB activity in RAW264.7 cells

The mouse macrophage cell line RAW264.7/Luc-P1 used in this study was established in our previous publication [22]. The RAW264.7/Luc-P1 cells were transfected with pELAM-1 and respectively seeded at the density of 4×10^5^ cells and allowed to grow overnight. Subsequently, the resulting cells were treated with lipopolysaccharide (LPS; the positive control) or the indicated materials for 5 h, harvested and analyzed using luciferase assays as described previously [22]. The luminescence was measured with an AutoLumat LB953 (Berthold Technologies, Bad Wildbad, Germany). The Mann-Whitney *U* test was used as a robust statistical comparison of NF-kB activity of the aqueous (MfA) and detergent (MfD) treated cells without prior distribution-related assumptions. The null hypothesis was that there is no difference between the distributions of *P* values of MfA and MfD treated cells, and MfD has a greater effect than MfA as the alternative hypothesis. The null hypothesis was rejected at p<0.005.

### Bioinformatics analysis of *Mycoplasma fermentans*

Preliminary ORF functional assignment was carried out by BLASTP [23] to search against the KEGG [24] bacterial protein database and predicted proteins of two recently available *M. fermentans* genomes (JER [25] and PG18 [26]) with the following criteria: bit score greater than 70, expectation value less than 10^−4^, percent identity greater than 30 and percent coverage of both query and hit sequences greater than 70. Using the same selection criteria, species conservation information was also recorded. Transmembrane domains of all ORFs were predicted by TMHMM [27]. Lipoproteins were predicted using LipoP [28] and homology search using previously-known *Mycoplasma* lipoproteins (P57, MALP-404, P78, P29, P48, P59, P68, P80, ORF700 and M161Ag) collected from NCBI Protein database [29]. Specifically, during LipoP prediction, any peptides containing predicted cleavage sites for signal peptidase II in the N-terminal region with log-odds score greater than zero was considered as potential lipoproteins. Continuous B-cell epitopes and protein antigenicity were predicted using COBEpro and ANTIGENpro respectively [30], [31]. The functional classification was performed by BLASTPGP [23] searching against the Cluster of Orthologous Groups (COG) dataset maintained by STRING database [32].

### Mollicutes phylogenetic analyses

In this study, *M. fermentans* M64 and 37 Mollicutes 16 S ribosomal gene sequences retrieved from NCBI were used for phylogenetic analysis (see Table S1). These sequences were aligned using MAFFT (version 6.847b) with the Q-INS-I setting [33]. A neighbor-joining phylogenetic tree was constructed using MEGA software package (version 5.05) [34] with Kimura's two parameter distance correction model and 1000 bootstrap replications.

## Results and Discussion

### 2-DE of M. fermentans

**Figure 1:**
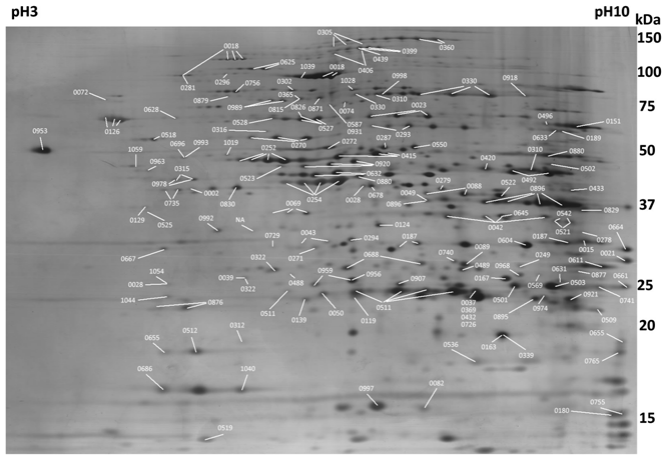
Representative 2-DE gel of *Mycoplasma fermentans* M64. A 100 µg of total proteins were separated by a 24 cm pH 3–10 linear IPG strip for the first dimension IEF and a 24×20 cm 11–13% gradient gel for the second dimension SDS-PAGE. After silver staining, an average of 700 spots can be detected on a 2-DE gel. Spots are numbered according to the matched ORFs.

Poor focusing on basic pH regions of 2-D gels was first observed when using typical passive rehydration of IPG strips. The p*I* calculation of all predicted ORFs reveals that about 77% of these proteins have theoretical p*I* greater than or equals to 8. Thus, rehydration followed by cup loading was generally employed in this study to achieve better resolution. The 2D gels (24×20 cm, pH 3–10) were produced in eight replicates and an average of 700 silver-stained spots per gel can be detected (see Figure 1). Regardless of protein isoforms or modifications, the ratio of spot number to predicted ORFs is similar to those in previous proteomic studies of other Mycoplasmas employing 2-DE approaches [35], [36], [37]. Under laboratory culture conditions, the bacteria can import nucleosides, amino acids and metabolites from the rich growth media, hence it is anticipated that only a minimum amount of proteins were expressed [38].

### Protein identification of *M. fermentans*

A total of 205 2D spots were successfully matched to 181 ORFs of *M. fermentans*. These spots were labeled with respect to their corresponding ORF numbers and shown in Figure 1. In addition, Table S2 summarizes these proteins along with their predicted functions, the predicted p*I*s and molecular masses as well as the conservation information in the two other sequenced *M. fermentans* strains (JER and PG18 [25], [26]) and also the four published human mycoplasmal pathogens: *M. genitalium* (mge), *M. hominis* (mho), *M. penetrans* (mpe) and *M. pneumoniae* (mpn).

Among the most intense spots in 2-DE of *M. fermentans*, several known proteins were identified including MfeM64YM0018 (D-xylulose 5-phosphate/D-fructose 6-phosphate phosphoketolase), MfeM64YM0126 (chaperone protein DnaK), MfeM64YM0252 (enolase), MfeM64YM0254 (elongation factor Tu), MfeM64YM0415 (glycyl-tRNA synthetase), MfeM64YM0511 (deoxyguanosine kinase) and MfeM64YM0896 (glyceraldehyde-3-phosphate dehydrogenase). Interestingly, some of them were also identified as the most abundant proteins in other mycoplasmas [35], [37]. These genes are highly conserved among Mycoplasma species and bacteria in general. They are commonly considered housekeeping genes that may be constitutively expressed, resulting in their high abundance inside the cells. On the other hand, the *M. fermentans*-specific hypothetical protein encoded by MfeM64YM0953 was also found to present as an abundant spot. On the *M. fermentans* chromosome, the MfeM64YM0953 gene is flanked by two housekeeping genes *nox* (NADH oxidase) and *argS* (arginyl-tRNA synthetase) on the same coding strand. The unknown protein expressed by the *M. fermentans*-specific MfeM64YM0953 may have a functional role in the *M. fermentans* physiology.

### Gene annotation of the identified *M. fermentans* ORFs

In order to functionally annotate the identified ORFs of *M. fermentans*, we performed a BLASTP search of the KEGG bacterial protein database. Each ORF was annotated according to the first BLASTP hit that satisfied the selection criteria. ORF that was matched only to another *M. fermentans* protein or without any BLAST match was interpreted as a *M. fermentans*-specific hypothetical protein. Among the 181 identified ORFs, 11 were conserved hypothetical proteins that were also found in bacteria other than *M. fermentans*, and 39 were *M. fermentans*-specific proteins. The annotation of the remaining 131 ORFs provides information about their regular functions and/or roles in *M. fermentans* life cycle.

Table S2 showed the number of putative orthologs of each identified *M. fermentans* M64 ORF found in the two other sequenced *M. fermentans* genomes and four published mycoplasmal human pathogens. Of the identified 181 ORFs from *M. fermentans* M64, MfeM64YM0672 is the only ORF that is absent in both *M. fermentans* PG18 and JER. MfeM64YM0049 and MfeM64YM0300 are the only ORFs that are absent in *M. fermentans* PG18. Compared to *M. fermentans* PG18, there were less *M. fermentans* M64 orthologs identified in *M. fermentans* JER. Among the 18 *M. fermentans* M64 ORFs which have no homologs in *M. fermentans* JER, there are two copies of *Mycoplasma* phage structural protein HtpA (MfeM64YM0587 and MfeM64YM0931), a cytosine-specific methyltransferase (MfeM64YM0826), a conserved hypothetical lipoprotein (MfeM64YM0714), two conserved hypothetical protein (MfeM64YM0249 and MfeM64YM0569) and 12 hypothetical ORFs.

**Figure 2:**
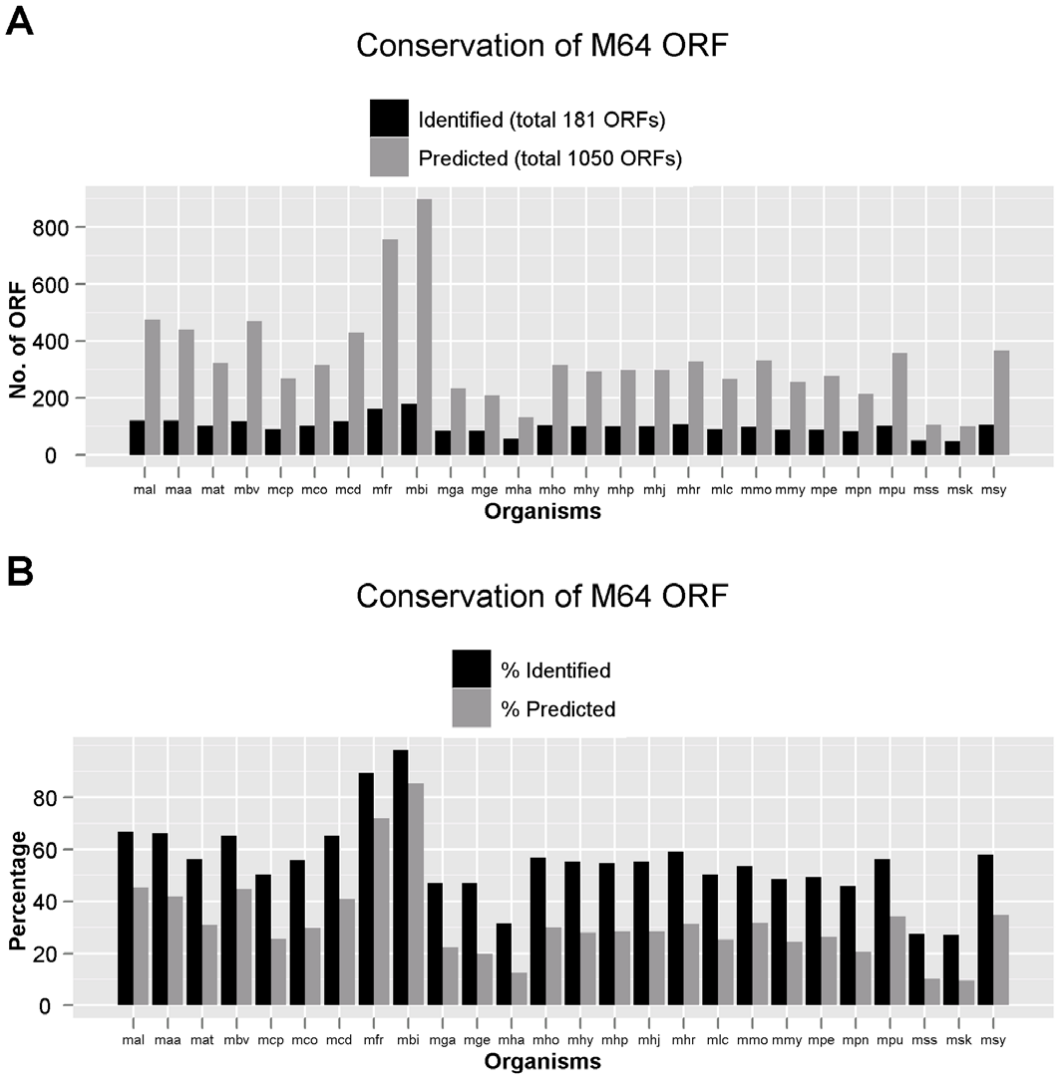
Proportion of *M. fermentans* M64 homologs in 26 mycoplasmal genomes. The three-letter KEGG organism codes were used in the x-axis. The full species names and the values used to plot the bar chart can be found in Table S3.

**Figure 3:**
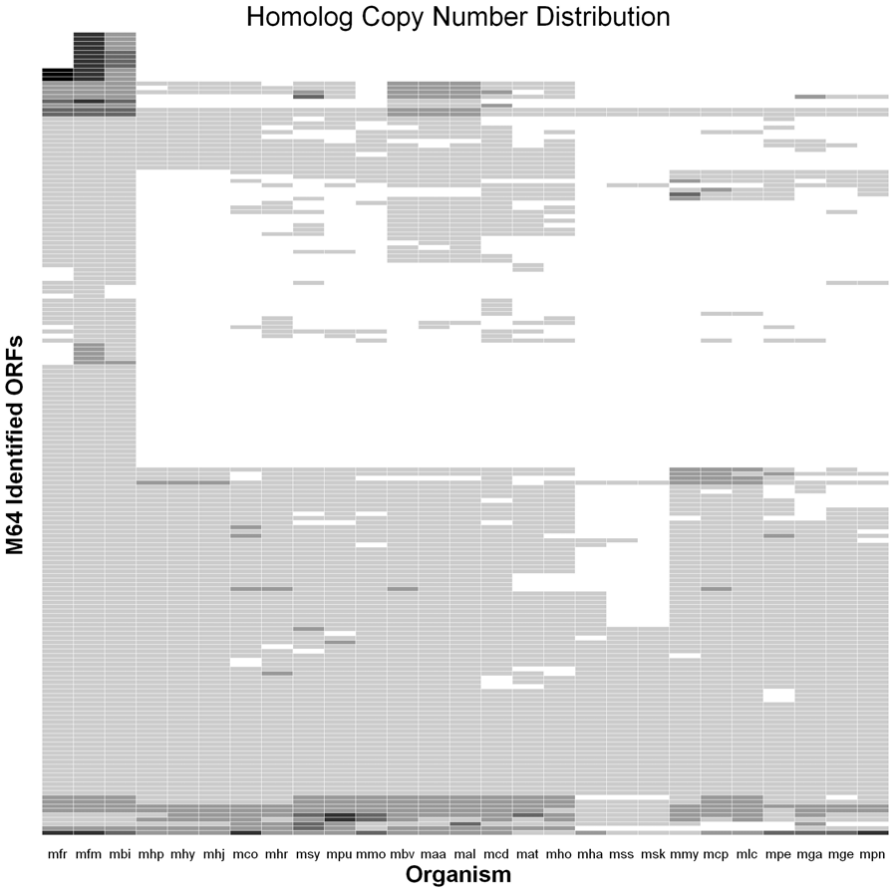
Heatmap representation of 181 *M. fermentans* M64 homologs in 26 mycoplasmal genomes. The number of putative homologs of a given *M. fermentans* M64 protein increases as color changes from white to black. Most homologs exist in single copy within a genome and are shaded in light gray. The x-axis and y-axis were hierarchically clustered based on the homology pattern in the analyzed genomes.

**Figure 4:**
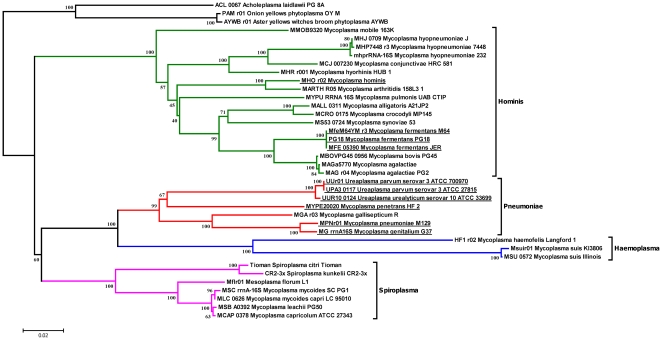
A representative phylogenetic tree for the selected Mollicutes species. The tree was constructed by neighbor-joining algorithm using 16 S ribosomal gene sequences from selected species of Mollicutes. The bootstrap values indicate the percentages of 1000 bootstrap replicates that support the branch. Most of the mycoplasmas can be categorized into four phylogenetic groups, namely pneumoniae, hominis, spiroplasma and haemoplasma. The 16 S sequence of *M. fermentans* M64, JER and PG18 are identical. All human pathogens were underlined. *Acholeplasma laidlawii*, AYWB phytoplasma and OY phytoplasma were used as the outgroup species in this analysis.

Predicted and identified proteome similarities between *M. fermentans* M64 and other sequenced mycoplasmal species were further illustrated in Figure 2 and Table S3. The distribution of putative homologs and paralogs of the 181 *M. fermentans* identified proteins in 26 mycoplasmal genomes were illustrated in Figure 3. Our results showed *M. fermentans* M64 shares the highest amount of both conserved identified and predicted proteins with *M. fermentans* PG18, followed by *M. fermentans* JER, and shares the least with *M. suis* and *M. haemofelis*. Predicted proteome conservation drops drastically outside the *M. fermentans* species, where *M. agalactiae*, *M bovis* and *M. crocodyli* shares close to 500 conserved ORFs with *M. fermentans* M64. *M.* fermentans shared the highest number of predicted homologs with *M. agalactiae* and *M. bovis* is in accordance with the results of phylogenetic analysis using 16 S ribosomal gene sequences (see Figure 4). The NJ tree shows that *M. fermentans*, *M. agalactiae*, and *M. bovis* were phylogenetically close to each other in the hominis group. However, the three species were known to infect different animal hosts despite their relatively closeness in phylogenetic distances. *M. fermentans* is a human pathogen causing respiratory illness and arthritis, whereas *M. agalactiae* infects sheep and goats and causes mastitis, arthritis, and pneumonia, and *M. bovis* PG45 causes respiratory disease, mastitis, and arthritis in cattle. The reptilian pathogen *M. crocodyli* is the third most similar mycoplasmal species compared with *M. fermentans* that causes exudative polyarthritis in crocodiles. Like *M. fermentans*, *M. hominis* is a member of the hominis group but shares little proteome similarities. Although *M. fermentans* and *M. hominis* are both human pathogens, they are known to colonize different part of the human body, with *M. fermentans* mainly isolated from the respiratory tract and *M. hominis* inhabits the female genital tract, hence the genomic make-up of these two species differs. Other known human mycoplasmal pathogens, such as *M. penetrans*, *M. pneumoniae* and *M. genitalium*, are members of the pneumonia group. They are phylogenetically distant to *M. fermentans* M64 and shares very little proteome homology.

Mycoplasmas are believed to be undergoing regressive/degenerate evolution and many ancestral genes were lost in the process, generating distinctive phylogenetic clades and species [39], [40]. The phylogenetically distant hominis, pneumonia and haemoplasma groups appear to be diverged very early and therefore their members are expected to have very diverse gene contents. There were a few reports suggesting the occurrence of horizontal gene transfer (HGT) in *Mycoplasma* species of the different phylogenetic groups sharing the same hosts and ecological niches [41], [42]. However, the genes involved in HGT were estimated to be around 3% to 18% between *M. gallisepticum* and *M. synoviae*, and between *M. agalactiae* and mycoplasmas of the mycoides cluster. Although *M. pneumoniae* and sometime *M. penetrans* shares the same ecologic niche as *M. fermentans* in the human respiratory tract, there was no sign of HGT between these respiratory mycoplasmas. Most of the homologs shared by the three species were conserved among other *Mycoplasmas* species and probably inherited from the last universal common ancestor.

### Functional categorization of *M. fermentans* ORFs

Of the 1050 predicted ORFs of *M. fermentans*, 590 predicted ORFs can be classified into 19 functional categories. Table 1 summarizes the assigned COG categories and relative percentages of identified ORFs within each category. The COG annotations of identified and predicted proteins were provided in Tables S2 and S4 respectively. The remaining 460 unclassified ORFs were denoted as COG-absent proteins. In this study, COG analysis of 181 identified proteins places 135 ORFs into 18 categories and 46 without COG annotation.

**Table 1:** Summary of the COG classification of the identified and predicted *M. fermentans* M64 ORFs.

COG ID	Description	No. of identified ORFs^a^	No. of predicted ORFs^b^	Identification ratio
INFORMATION STORAGE AND PROCESSING				
J	Translation, ribosomal structure and biogenesis	33	102	32.35%
K	Transcription	8	31	25.81%
L	DNA replication, recombination and repair	11	93	11.83%
CELLULAR PROCESSES				
D	Cell division and chromosome partitioning	3	13	23.08%
V	Defense mechanisms	1	23	4.35%
T	Signal transduction mechanisms	0	7	0.00%
M	Cell envelope biogenesis, outer membrane	4	18	22.22%
U	Intracellular trafficking, secretion, and vesicular transport	1	23	4.35%
O	Posttranslational modification, protein turnover, chaperones	8	17	47.06%
METABOLISM				
C	Energy production and conversion	7	22	31.82%
G	Carbohydrate transport and metabolism	19	62	30.65%
E	Amino acid transport and metabolism	7	30	23.33%
F	Nucleotide transport and metabolism	12	23	52.17%
H	Coenzyme metabolism	3	12	25.00%
I	Lipid metabolism	1	9	11.11%
P	Inorganic ion transport and metabolism	2	28	7.14%
Q	Secondary metabolites biosynthesis, transport and catabolism	1	3	33.33%
POORLY CHARACTERIZED				
R	General function prediction only	16	67	23.88%
S	Function unknown	3	43	6.98%
OTHERS				
COG-absent		46		10.00%

a The COG assignment of each identified protein can be found in Table S2.

b The COG assignment of each predicted protein can be found in Table S4.

For functions in the Information Storage and Processing, several key proteins for transcriptional process were identified, including the universally conserved ribonuclease III (Rnc) for RNA processing, α and β subunits of DNA-directed RNA polymerase (RpoA and RpoB), transcription termination/antitermination factor NusG, transcription elongation factor NusA and GreA. Major protein components for translation and ribosome assembly were also identified, including the translation initiation factor IF-2 and IF-3, essential elongation factors Tu, Ts and G, aminoacyl-tRNA synthetases and ribosomal proteins. In addition, essential proteins for DNA replication were obtained, including the DNA polymerase III tau/gamma (DnaX) and beta (DnaN) subunits, DNA gyrase A (GyrA) and B (GyrB) subunits, single-strand binding protein and DNA topoisomerase I. Previously, it has been shown that mycoplasmas have a minimal set of genes for DNA repair; the nucleotide excision repair system is perhaps the most important repairing pathway in mycoplasmas [43]. The beta subunits of the ABC exonuclease (UvrB) was the only identified proteins for DNA repair. Identification of the transcriptional accessory factor Tex may suggest certain level of control in *M. fermentans* pathogenesis. Tex was first discovered in *Bordetella pertussis* as an essential protein for expression of toxin genes [44], and is found to be involved with pathogen fitness of *Streptococcus pneumoniae* [45]. In addition, crystal structure of Tex has been recently determined in *Pseudomonas aeruginosa*, and the authors suggested probable involvement of transcription regulation in both eukaryotic and prokaryotic organisms [46].

In general, a low number of proteins were identified from the Cellular Process subcategories. Such poor identification is similar in previous proteome analyses of other mycoplasmas [35], [36], [37]. One exception is the “post-translational modification, protein turnover and chaperones" category where 50% of predicted proteins were identified, including chaperone protein (DnaK), ATPase subunit of ATP-dependent Clp protease (ClpB), ATP-dependent protease (Lon) and endopeptidase O (PepO). PepO is structurally similar to the mammalian neutral endopeptidase and studies have found evidence in host cell invasion and membrane lysis [47].

**Figure 5:**
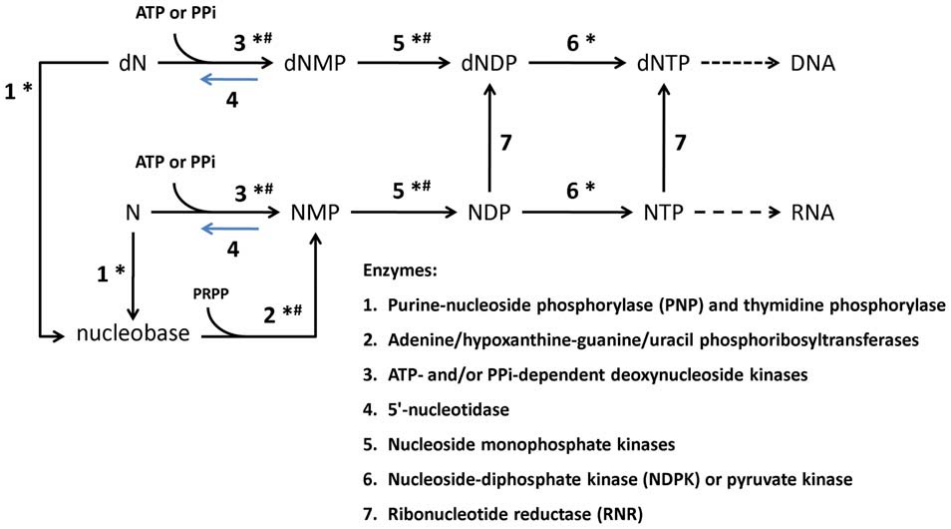
An adaption of the schematic representation of the nucleoside salvage pathway by Wang, et al., 2001 [**50**]. Substrates/products: dN/N (deoxy)nucleoside; dNMP/NMP (deoxy)nucleoside monophosphate; dNDP/NDP (deoxy)nucleoside diphosphate; dNTP/NTP (deoxy)nucleoside triphosphate; PRPP 5-phosphoribosyl diphosphate; ATP adenosine 5′-triphosphate; PPi pyrophosphate. Identified enzymes were labeled with asterisk (*) and predicted enzymes but not identified were labeled with hash (#), details can be found in Table S4.

**Figure 6:**
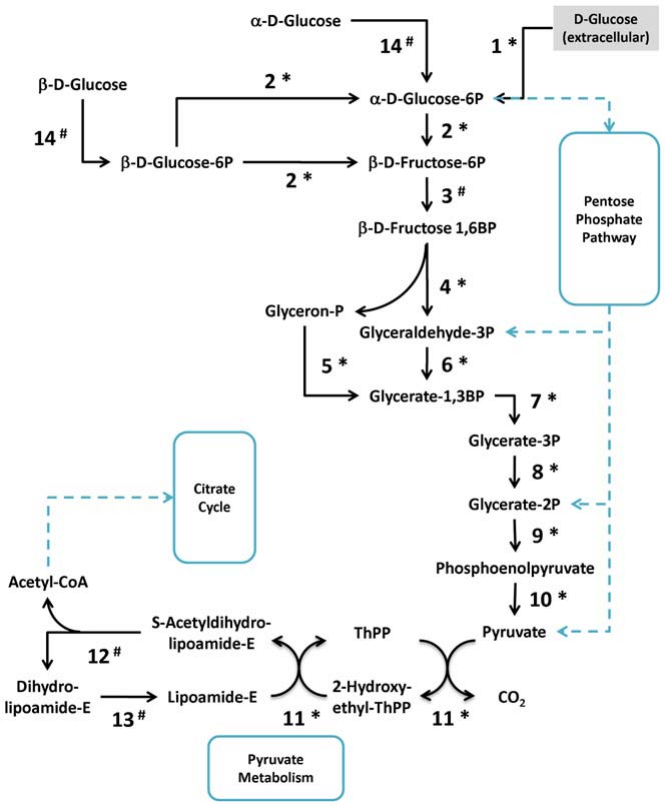
A representative scheme of *M. fermentans* proteins involved in the glycolysis pathway. The identified enzymes were labeled with asterisk (*) and the predicted enzymes but not identified in this study were labeled with hash (#). Further details were summarized in Table S5.

Protein identification differs substantially among the subcategories of Metabolism. Seven proteins of “energy producing and conversion" were identified, including the key enzymes such as subunits of the ATP synthase and pyruvate dehydrogenase. Twelve proteins of “nucleotide transport and metabolism" were identified that function in the nucleoside salvage pathways (see Figure 5 and Table S5). Mycoplasmas are incapable of de novo synthesis of purine and pyrimidine bases and depend heavily on the external supplement of purine/pyrimidine bases and nucleosides [48], [49]. As shown in Table S5, most of the enzymes that participate in the salvage pathways were predicted in *M. fermentans*, except for some nucleoside/deoxynucleoside kinases. This is not uncommon in mycoplasmas and it is likely that the two deoxyguanosine kinases, MfeM64YM0510 and MfeM64YM0511, may have broad substrate specificity as observed in *M. mycoides* subsp. *mycoides* SC [50]. In 2002, some metabolic kinases, including 6-phosphofructokinases (6-PFKs), phosphoglycerate kinases (PGKs), pyruvate kinases (PKs), and acetate kinases (AKs) were shown to possess nucleoside diphosphate kinase (NDPK)-like activity in several *Mycoplasma* species. This suggests functional compensation of the household *ndk* gene [51]. Identification of these proteins demonstrates that *M. fermentans* utilizes the salvage enzymes similar to other mycoplasmas to recover nucleosides as carbon and energy sources [50], [52]. Nineteen proteins of “carbohydrate transport and metabolism" were obtained, including the major components for glycolysis and pentose phosphate pathway (see Figure 6 and Table S6). Seven proteins of “amino acid transport and metabolism" were identified, including the arginine deiminase (ArcA), ornithine carbamoyltransferase (ArcB) and carbamate kinase (ArcC). These proteins represent the complete set of enzymes in the arginine dihydrolase pathway (ADI), which is considered the major ATP production pathway other than glycolysis in arginine-fermenting mycoplasmas [48]. Besides the three *M. fermentans* strains, *M. crocodyli*, *M. penetrans*, *M. arthritidis*, *M. pneumoniae* and *M. hominis* are the only six of the total 27 sequenced mycoplasmas to possess all three genes (*arcABC*). Presence of both glycolysis and the arginine deiminase pathway indicates that *M. fermentans* could utilize both pathways for ATP production. Furthermore, co-existence of these two pathways supports a previous finding that glucose metabolism has little or no effect on arginine utilization in *M. fermentans* [53].

### Identification and prediction of membrane proteins

The membrane proteins and lipoproteins were important for cell wall-less bacteria such as *M. fermentans* to communicate with the environment, and some of them have been suggested as virulence factors or immunomodulatory agents [7]. Unlike the soluble cytosolic proteins analyzed in 2-DE, membrane proteins and lipoproteins of *M. fermentans* rely on detergent extraction to be prepared [21]. In this study, lipoproteins/membrane proteins were identified using Triton X-114 extraction/SDS-PAGE and mass spectrometry. The resulted proteins were shown together with other identified proteins in Table S2.

**Figure 7:**
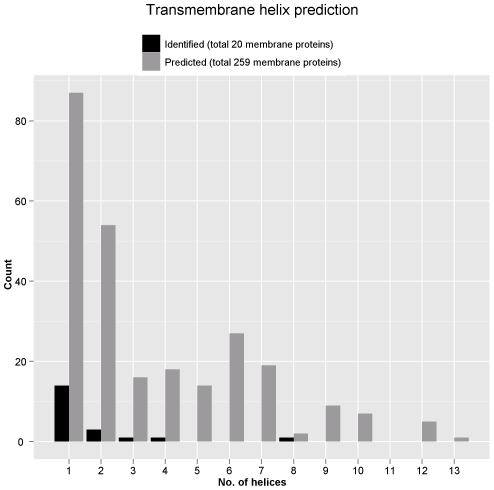
The number of transmembrane helices predicted using TMHMM. The prediction was performed on all *M. fermentans* M64 predicted and identified proteins. Of the 1050 total predicted ORFs, 791 (75%) have no predicted TMD. Whereas 161 of 181 (89%) identified ORFs have no predicted TMD.

In addition, bioinformatics analyses using TMHMM algorithm were performed to predict proteins carrying transmembrane domain (TMD). The program predicts 259 membrane proteins from the total *M. fermentans* M64 ORFs, and 20 were identified in this study (summarized in Figure 7 and Table S2). Most of the identified membrane proteins from both 2-DE and Triton X-114 extraction/SDS-PAGE contain only one TMD. Nevertheless, the MfeM64YM1059 that contains eight predicted TMDs was solely identified from 2-DE.

Among the identified membrane proteins, MfeM64YM1059 has the most number of TMDs and is an ABC transporter permease protein. MfeM64YM0048 has four TMDs and is predicted to function as a glycosyltransferase, and other similar homologs (30∼31% amino acid identity) outside the *M. fermentans* family were found in *Bifidobacterium longum* and *B. bifidum*. MfeM64YM0981 has two predicted TMDs and was annotated the ATP-binding protein of an unspecified sugar ABC transport system. The cell division protein FtsH (MfeM64YM1060) also has two TMDs. Four proteins, MfeM64YM0281, MfeM64YM0330, MfeM64YM0621 and MfeM64YM0879 were predicted to have only one TMD. MfeM64YM0281 encodes the substrate-binding component of an oligopeptide ABC transporter system. The predicted protein encoded by MfeM64YM0330 resembles the 81-kDa surface membrane protein (P80) of *M. agalactiae* encoded by *ma-mp81* [54]. Sequence analysis of MfeM64YM0330 shows amino acid sequence homology with other putative proteins and lipoproteins of 23 other *Mycoplasma* genomes (see Table S7). MfeM64YM0879 and MfeM64YM0880 were annotated as the hominis P80-like and P60-like hypothetical protein respectively. In *M. hominis*, the genes encoding the 80 kDa protein (P80), 60 kDa protein (P60), and histidine triad nucleotide-binding protein (HinT) are organized in a *hitABL* operon [55]. Both P80 and P60 of *M. hominis* possess signal peptides that allow the proteins to translocate to the membrane and form P60–P80 surface protein complex. The cytoplasmic HinT protein interacts with the complex by binding to P80. Upon activation due to environmental changes, the signal peptidase will process the membrane-bound P80 into a soluble form and release it into the extracellular environment [56]. With sequence homology search and organizational conservation, the *hitABL* operon was found to be conserved in 21 sequenced *Mycoplasma* genomes (see Table S7). There are insignificant sequence similarities between the *M. hominis* P80 and the *M. agalactiae* P80 lipoproteins (see Figure S1). Since most sequenced mycoplasmal genomes carry both P80 proteins and the alignment result showed distinctively different amino acid composition, the two P80 proteins are clearly unrelated but were historically named P80 simply due to their detected molecular weights. MfeM64YM0621 is a LemA protein, this family of proteins was previously reported to have a predicted N-terminal transmembrane helix and an unknown function [57]. Other identified TMD-containing proteins, including MfeM64YM0115, MfeM64YM0190, MfeM64YM0246, MfeM64YM0336, MfeM64YM0358, MfeM64YM0360, MfeM64YM0399, MfeM64YM0411, MfeM64YM0439, MfeM64YM0672, MfeM64YM0902, and MfeM64YM0953, are *M. fermentans*-specific proteins and unable to be assigned with any functional category, suggesting further investigation to illustrate their amphiphilic features.

### Identification of lipoproteins as putative virulence factor

**Figure 8:**
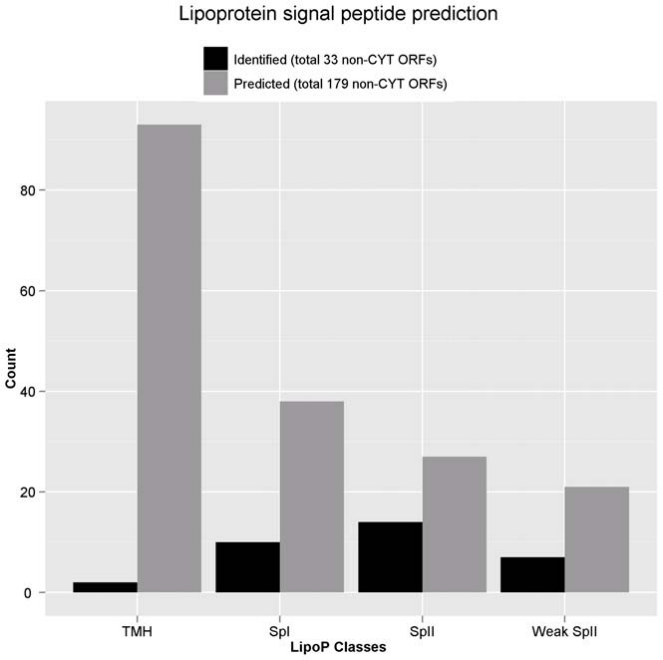
Signal peptide-containing protein identification by LipoP. The prediction was performed on all predicted and identified proteins of *M. fermentans* M64. ORFs predicted as cytosolic proteins (CYT) were excluded in the chart.

**Figure 9:**
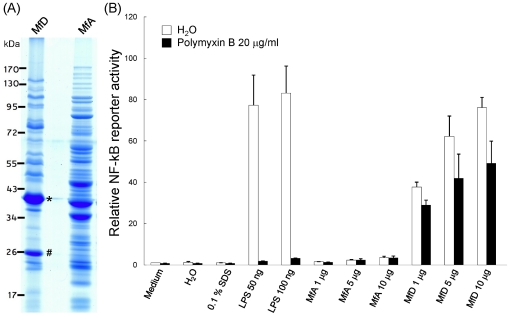
NF-kB activation of proteins from Triton X-114 extraction of *M. fermentans* M64. (A) SDS-PAGE analysis of proteins from Triton X-114 fraction (MfD) and aqueous fraction (MfA) of *M. fermentans* M64. Proteins labeled with asterisk (*) or hash (#) were the previously known MALP-404 and P29 respectively. (B) NF-kB activating ability of different controls and 1, 5, 10 µg of MfD or MfA fractions in 0.1% SDS solution. LPS is a positive control of NF-kB activation. Polymyxin is an antagonist of LPS. Addition of polymyxin is to exclude the contamination of LPS in the measured samples. In both H_2_O and polymyxin, the p-values of significance from the one-tailed Mann-Whitney *U* test are both <0.005.

Bacterial lipoproteins are often recognized as virulence factors due to its immunogenic properties. Membrane-localized lipoproteins often play an important role in the interaction between the bacteria and host cells. Not only do they involve in bacterial adhesion and coaggregation, lipoproteins also have been shown to stimulate the release of pro-inflammatory cytokines. In this study, several *M. fermentans* predicted lipoproteins were identified, including a number of known lipoproteins, from the Triton X-114 extraction. Using LipoP [28], 48 total *M. fermentans* M64 ORFs were predicted to contain type II lipoprotein signal peptides and 38 ORFs contain type I signal peptide (Figure 8). Among the 48 predicted lipoproteins, 21 of them (44%) were identified in this study and their N-terminal signal peptide sequences along with possible cleavage sites were summarized in Table 2. Furthermore, the NF-kB activation of Triton X-114 extraction was measured using a Luciferase reporter assay to determine whether such detergent fraction contains potent NF-kB-activating lipoproteins (Figure 9). Our results showed that the proteins in Triton X-114 extraction showed a significant NF-kB activation activity in mouse macrophage cells compared to the aqueous fraction. (p = 0.001082).

**Table 2:** List of identified lipoproteins from *Mycoplasma fermentans* M64.

ORF ID^a^	Cleavage site	Amino acid at Pos +2	Predicted signal peptide^b^	Conservation^c^
MfeM64YM0021^#+^	30	G	MRRKSMNKKFLKLGSIAGILSFAPVAISAG*CGSKG	2
MfeM64YM0039^+^	23	N	MKKKILLSNLIWSLPLLPLSTLS*CNNQT	2
MfeM64YM0281^+^	28	G	MNKKRRLLWSFAPLATLAAFAPVAISAS*CGRKG	5
MfeM64YM0300^+^	30	Q	MKEKYMKKKIKLSLIFSSVTAGVLPLISSQ*CQNTT	1
MfeM64YM0330^#+^	26	G	MKKANKLIISLGTAFAALSPVVLSVG*CGGGG	4
MfeM64YM0331^+^	26	Q	MKFKNNTLNFCLNTIVPSMAIVVCSS*CQSKD	2
MfeM64YM0380^#+^	25	F	MKKSKKFMKFMAIGTIVPLASLTTS*CFGPI	2
MfeM64YM0336^+^	29	N	MNKKMKFKKLMFIAASTLALGVAPAITMS*CNFYI	2
MfeM64YM0433^#+^	23	Q	MKKIYKFIVASSSIFALGSISAG*CQYQP	2
MfeM64YM0451^#+^	25	F	MKKSKKFMKFMAIGTIVPLASLTTS*CFGPI	2
MfeM64YM0519	22	V	MFKKKLLISSLAVASFLPVSVV*CVSCG	2
MfeM64YM0616^+^	23	K	MKKIKMILTLSSLTTCLPIIATS*CKNEK	2
MfeM64YM0688	24	G	MKLKKIFKFGALLTSPIMLVPIVS*CGKNQ	2
MfeM64YM0714^+^	29	G	MSNFKKTFKKIAIGFATLAAPVTALATIS*CGTTE	4
MfeM64YM0802^#+^	25	F	MKKSKKFMKFMAIGTIVPLASLTTS*CFGPI	2
MfeM64YM0846^#+^	25	F	MKKSKKFMKFMAIGTIVPLASLTTS*CFGPI	2
MfeM64YM0871^#^	22	G	MKKNKIFNSLLLTPLIITPITS*CGSKP	1
MfeM64YM0895^+^	25	G	MKKNLLKLTGIIAPVITAIPFVASS*CGSKD	2
MfeM64YM0978^#+^	24	G	MKKSKKILLGLSPIAAVLPAVAVS*CGNND	8
MfeM64YM0984^#+^	26	T	MKKRKFIFALSSLTTAFLATPLISSS*CTNAD	2
MfeM64YM1013^+^	24	L	MKKFKWLLVPSVIGVLSTPMISVS*CLGNQ	2

a ORFs with homology to known lipoproteins deposited in NCBI were marked with hash (#). ORFs identified in the protein fraction capable of elicit NF-kB activation were marked with plus (+).

b Cleavage sites were marked with asterisk (*). Positively charged arginine (R) and lysine (K) residues in the N terminus were underlined. Cysteine (C) residues immediately followed the predicted cleavage site were shadowed.

c Number of mycoplasmal complete genomes containing the particular *M. fermentans* M64 homologue.

By comparing with known mycoplasmal lipoprotein sequences, seven of the 21 identified lipoproteins were similar to previously reported lipoproteins as indicated in Table 2. MfeM64YM0021 is the phase variant surface lipoprotein P29 [58] and its expression was found to mediate the adherence of *M. fermentans* to host cells [59]. MfeM64YM0281 (*oppA*) is distantly similar to the P100 (OppA) protein in *M. hominis* where both are encoded by an *opp* operon. Besides functioning as a peptide-binding domain of an oligopeptide permease system (Opp), previous studies showed that P100 mediated adherence of *M. hominis* to host cells and also served as the major ATPase on the surface of mycoplasmal cells, and by inducing ATP release and hydrolysis, it caused the apoptosis of HeLa cells *in vitro* [60]. MfeM64YM0330, as previously described, is similar to the surface membrane lipoprotein P80 of *M. agalactiae* [54]. MfeM64YM0433 is the ortholog of a phosphonate transport system substrate-binding protein, P37. P37 was first sequenced in *M. hyorhinis* [61] and the protein itself and the bacterium *M. hyorhinis* have been associated with several kinds of cancers [62]. In 2009, Sippel et al. has resolved the crystal structure of *M. hyorhinis* P37 [63]. Based on the newly discovered 3D structure, it is now designated the extracytoplasmic thiamine-binding lipoprotein, where it may be involved in sequester the thiamine from the environment into the bacteria, hence depleting the thiamine source for host cells, thus weakening them and making them prone to infection. The MfeM64YM0451, MfeM64YM0802 and MfeM64YM0846 lipoproteins share identical nucleotide and amino acid sequences. We confirmed that these three genes all encode the P57 lipoprotein, whereas the MfeM64YM0380 encodes the alternative P57′ protein product. The *p57* and *p57′* alleles were first identified exclusively in the ICEF (integrative conjugal elements of *M. fermentans*) fragments of *M. fermentans* by Lu [64]. Calcutt et al. showed *M. fermentans* isolates M39B and M70B have a mixture of the *p57* and *p57′* alleles, whereas SK5 and SK6 contained only the *p57′* allele [65]. The protein similarity (based on BLAST identity score) between the two allelic forms in *M. fermentans* M64 is very high (97.5%). The peptide fragments identified by MASCOT resides in the homologous regions of both alleles, therefore further experiment is suggested to confirm the presence of one or both allelic forms in the *M. fermentans* M64 proteome. MfeM64YM0871 shares a 45% protein sequence similarity to ORF700, which was regarded as an integrative conjugal elements of *M. fermentans* PG18 (ICEF) in a previous study [65], and like ORF700, MfeM64YM0871 has a transposase gene (MfeM64YM0869) encoded upstream. MfeM64YM0978 is the macrophage activating lipoprotein (MALP-404) precursor which has drawn lots of attention for its ability in host immune activation [7], [9]. MfeM64YM0984 is the phase variable surface lipoprotein P78 precursor of *M. fermentans* [66]. P78 was proposed as a component of a putative ABC transporter system [67].

Among the previously unknown lipoproteins in *M. fermentans*, MfeM64YM0039 is annotated as BspA-like leucine-rich repeat protein. BspA was first identified in a human parasite, *Tannerella forsythia* (formally *Bacteroides forsythus*), and was known to bind extracellular matrix and is immunogenic [68], which raises the possibility that MfeM64YM0039 may participate in *M. fermentans* colonization. MfeM64YM0714 is annotated as conserved hypothetical lipoprotein that has homologs in *M. crocodyli*, *M. hyorhinis* and *M. pulmonis*. Finally, the remaining eight identified lipoproteins were *M. fermentans* species-specific and remain functionally unknown.

Using COBEpro and ANTIGENpro, the antigenicity and the presence of continuous B-cell epitopes of the 20 identified lipoproteins were predicted and summarized in Table S8. The results showed some of the lipoproteins have three top predicted B-cell epitopes clustered together, including MfeM64YM0039, MfeM64YM0331, MfeM64YM0519, MfeM64YM0616, MfeM64YM0978 and MfeM64YM1013. Five proteins (MfeM64YM0336, MfeM64YM0380, MfeM64YM0451, MfeM64YM0802 and MfeM64YM0846) have all three epitopes found at different locations, indicate that they might have multiple immunogenic sites whereas others only have one major site. MfeM64YM0330 (P80) and MfeM64YM0281 (OppA) are the two most likely antigenic proteins. Other previously reported lipoproteins such as P29 (MfeM64YM0021), P37 (MfeM64YM0433), P57 (MfeM64YM0451, MfeM64YM0802, and MfeM64YM0846), P57′ (MfeM64YM0380), P78 (MfeM64YM0984) and MALP-404 (MfeM64YM0978) also have antigenicity likelihood scores greater than 0.5. Four lipoproteins (MfeM64YM0871, MfeM64YM0688, MfeM64YM0895 and MfeM64YM0519) have scores less than 0.5 and were annotated as *M. fermentans*-specific lipoprotein. This dataset offers possible candidates for diagnostic tests or synthetic peptide vaccine development. Further study of the roles of novel identified lipoproteins in *M. fermentans* virulence and pathogenesis is recommended.

### Overall comparison of proteomic profiles with other Mycoplasma species

In the past decade, several Mycoplasma proteomes have been reported including human and non-human pathogens. The identified protein datasets from these studies were summarized in Table 3. Although the ratio of identified proteins varies significantly among diverse species, *i.e.* 90% in *M. pneumoniae* (689 predicted ORFs) and 5% in *M. synoviae* (659 predicted ORFs), the genome size and culture conditions may be the contributing factors. As reviewed by Catrein and Herrmann [69], a total of 620 distinct proteins of human pathogen *M. pneumonia* were reported from four different studies, which respectively identified 41, 305, 557 and 411 proteins [70], [71], [72], [73]. Whereas all these studies were carried out using bacteria grown under host-free conditions, diverse sample preparation and identification approaches help maximize the identification coverage. *M. pneumoniae* is a relatively small human pathogen with 689 predicted ORFs of which 207 have homologs in *M. fermentans*. In this study, 81 (39%) proteins homologous to *M. pneumoniae* were identified. On the other hand, another human pathogen, *M. penetrans* (1037 ORFs), has a similar genome size to *M. fermentans* (1050 ORFs) and their identification coverage are close, 14% and 17% respectively. Of the reported 153 *M. penetrans* proteins, 78 have homologs in *M. fermentans* and 52 (67%) of them were identified in this study. Study of *M. genitalium*, the smallest human pathogen with 475 ORFs, also reported similar identification coverage (18%) to *M. fermentans*. Physiological distribution and pathological significance of these four human pathogens are quite different. The conserved or species-specific proteins of *M. fermentans* may provide valuable information regarding its virulence, optimal growth conditions and host specificity. Table S2 summarized the conservation of the identified *M. fermentans* proteins among four other human Mycoplasma pathogens.

**Table 3:** List of available *Mycoplasma* proteome datasets.

Organisms^a^	Host	No. of predicted ORFs	No. of identified proteins	Identification coverage (%)	Data source
***M. pneumoniae***	Human	689	620	89.99%	[69], [70], [71], [72], [73]
*M. mobile*	Fish	633	557	87.99%	[77]
*M. gallisepticum*	Chickens and turkeys	763	487	63.83%	[78]
*M. agalactiae* 5632	Sheep and goats	813	453	55.72%	[79], [80]
*M. agalactiae* PG2	Sheep and goats	742	194	26.15%	[79], [80]
*M. hyopneumoniae* J	Pigs	657	164	24.96%	[81], [82]
*M. hyopneumoniae* 7448	Pigs	657	159	24.20%	[81], [82]
*M. hyopneumoniae* 7422^b^	Pigs	N.D.	158	N.D.	[81], [82]
***M. genitalium***	Human	475	86	18.11%	[36]
***M. fermentans*** ** M64**	Human	1050	181	17.24%	This report
***M. penetrans***	Human	1037	153	14.75%	[35]
*M. synoviae*	Chickens and turkeys	659	31	4.70%	[83]

a Human pathogens are highlighted in bold font.

b Whole genome sequence of *M. hyopneumoniae* 7422 is not available. Thus, values of total ORF number and identification coverage are not determined (N.D.).

To explore the functional significance of the identified proteins among diverse mycoplasmas, the distribution analysis of the identified and predicted proteins in COG categories of 11 Mycoplasma species in NCBI has been carried out and results were summarized in Figure S2A to F. Overall, the number of identified proteins in each COG category varies greatly among species, probably due to the varied numbers of total identified proteins. However, certain categories have relatively high numbers of identified proteins even in species with low identification coverage. These COG categories include Information Storage and Processing (J and K, Figure S2A), Cellular Processes (O, Figure S2C) and Metabolism (C, F and G, Figure S2D). These categories coincide with the COG categories that have high identification ratios in *M. fermentans* discussed in section 3.4, implying these proteins and pathways are essential for Mycoplasma species. Further examination of the mycoplasmal proteins in these categories, we found that many are encoded by housekeeping genes such as elongation factors, ribosomal proteins, chaperon proteins and enzymes participating in the glycolysis and pentose phosphate pathways. Ubiquitous housekeeping proteins are maintained in the cells between 10^5^ to 10^6^ copies and thus are sufficiently abundant to be detected in most proteomics techniques [74]. Presence of these universally identified proteins indicates their functional importance for the survival of the mycoplasmal cells in host-free environment. For example, the pyrimidine and purine-nucleoside phosphorylases which are important in the nucleoside salvage pathways are among the most identified proteins in mycoplasmal proteomics studies.

In several studies, several COG categories exhibited low identification numbers among several mycoplasmas, including proteins involve in Signal transduction mechanisms (T), Defense mechanism (V), Cell envelope biogenesis, outer membrane (M) and several Metabolism subcategories (see Figure S2B, S2C and S2E). This is likely due to the low-abundance of these proteins and the improvement in the sample preparation may help to increase the identification numbers.

On the other hand, absence of proteins in several categories may also be due to the lack of external stimulation from and interaction with the host cells, giving that certain proteins were not necessary during host-free cultivation. Especially in *M. pneumoniae* and *M. mobile*, where overall identification ratios are high, there are still a number of missing proteins in the subcategories of Transcription (K), Defense mechanism (V) and General function prediction only (R) along with no COG assignment. It is also possible that different biological pathways could be triggered under bacterial growth under host-free culture with rich medium. Like the other human pathogen *M. penetrans*, *M. fermentan*s M64 also has a relatively large genome and proteome. Both species have a large number of identified proteins in Transcription (K), Intracellular trafficking, secretion, and vesicular transport (U) and proteins with unknown COG functions compared with other mycoplasmal genomes. In addition, they have similar ratios of identified proteins in most of categories, implying these two mycoplasmas may have similar adaption in environment. Our results provided a basic knowledge of *M. fermentan*s proteins involved in the host-free cultivation. Further studies of this microorganism as well as other Mycoplasma species co-cultured with host cells are suggested.

### Conclusion

*M. fermentans* is a commonly found pathogen in human respiratory tract. Previously, whole genome sequencing data of two *M. fermentans* strains, PG18 and JER, have been deposited into NCBI database [25], [26]. In order to have a better understanding of this bacterium, we have recently completed the genome sequencing of *M. fermentans* M64 isolated from a local patient [15]. In the present study, we provide the first proteome description of *M. fermentans* as well as the *in silico* comparison of the identified 181 proteins with other completely sequenced *Mycoplasma* species, which was summarized in Table S2. Many proteins involved in essential metabolic pathways were identified, *i.e.* the nucleoside salvage pathways (KEGG pathway map00230 and map00240), the arginine dihydrolase pathway (map00330) and the glycolysis (map00010) and pentose phosphate pathway (map00030). Furthermore, our preliminary data showed that the lipoproteins from the Triton X-114 extraction of *M. fermentans* were able to induce the NF-kB activation in mouse macrophage cells, which may play important modulation in the host interaction of this pathogen. Currently, only one *M. fermentans* lipoprotein (MALP-404) has been intensively demonstrated to participate in the TLR-dependent immune response. Our identification of novel lipoproteins will add fundamental information to confer the molecular basis to study the pathogenesis and immunomodulatory roles of *M. fermentans* in human diseases.

Genomes of mycoplasmas were shaped by gene deletion events over time, which results in reduction in genome size [75]. Our comparative analysis in genomic scale showed high similarity exists among strains of *M. fermentans*, but considerable difference with other Mycoplasma species. Such observed genome content flexibility among different species is an evolutionary adaptive mechanism allowing members of this genus to infect a wide range of multicellular organisms and at the same time exhibit a strict host, organ and tissue specificity due to its limited genetic information [76]. The present study provides in-depth and unique information regarding the proteome of the *M. fermentans* for further functional studies of this small yet clinically important pathogen. The identified *M. fermentans*-specific proteins are potential candidates for diagnostic markers and vaccine development.

## Supporting Information

Figure S1 **Multiple sequence alignment of the agalactiae-P80 and hominis-P80 mycoplasmal lipoproteins.** The hominis-P80 cluster contains proteins similar to the M. hominis P80 and the agalactiae-P80 otherwise. Sequence conservation was shown in a gradient from blue (the most conserved), black to red (the least conserved).
(TIF)

Figure S2 **COG functional profile of the 11 mycoplasmal species.** The distribution of COG of total, identified and predicted-only proteins were shown as black, red and blue bars respectively. The organism names of human pathogens were labeled in red. A) COG classes related to Information Storage and Processing (J, K and L categories); B) and C) Cellular Processes (D, V, T, M, U and O categories); D) and E) Metabolism (C, G, E, F, H, I, P and Q categories) and F) Others (R, S and no COG assignment).
(PDF)

Table S1 **List of Mollicutes species used in 16 S phylogenetic analysis.**
(DOC)

Table S2 **List of the** ***M. fermentans*** **M64 proteins identified in this study.** The summary table includes the protein annotation, MASCOT scores, predicted p*I*s and molecular masses, COG classification, TMHMM and LipoP prediction results, KEGG orthology (KO) and pathway information.
(XLS)

Table S3 **Conservation of** ***M. fermentans*** **M64 proteins conserved across the fully sequenced Mycoplasma species.** The values showed the number and percentage of identified and predicted *M. fermentans* M64 proteins that are homologous with species in each row. The two percentage columns were colored according to the level of conservation, ranging from dark red (highest conservation) to dark green (lowest conservation).
(DOC)

Table S4 **COG annotations of the identified and predicted** ***M. fermentans*** **M64 proteins.**
(XLS)

Table S5 **List of enzymes that participate in the nucleoside salvage pathway.**
(DOC)

Table S6 **List of enzymes that participate in the glycolysis pathway.**
(DOC)

Table S7 **List of gene locus IDs that were used in the agalactiae-P80 and hominis-P80 lipoprotein multiple sequence alignment.**
(DOC)

Table S8 **Predicted protein antigenicity and continuous B-cell epitopes of identified lipoproteins from** ***Mycoplasma fermentans*** **M64.** The data was sorted by the predicted antigenicity likelihood of the lipoproteins.
(DOC)
