# Supplementary figures and images for: Proteomics Characterization of Cytoplasmic and Lipid-Associated Membrane Proteins of Human Pathogen Mycoplasma fermentans M64
Source: PLoS One. 2012 Apr 20;7(4):e35304. doi: 10.1371/journal.pone.0035304 (PMC3335035; doi:10.1371/journal.pone.0035304)

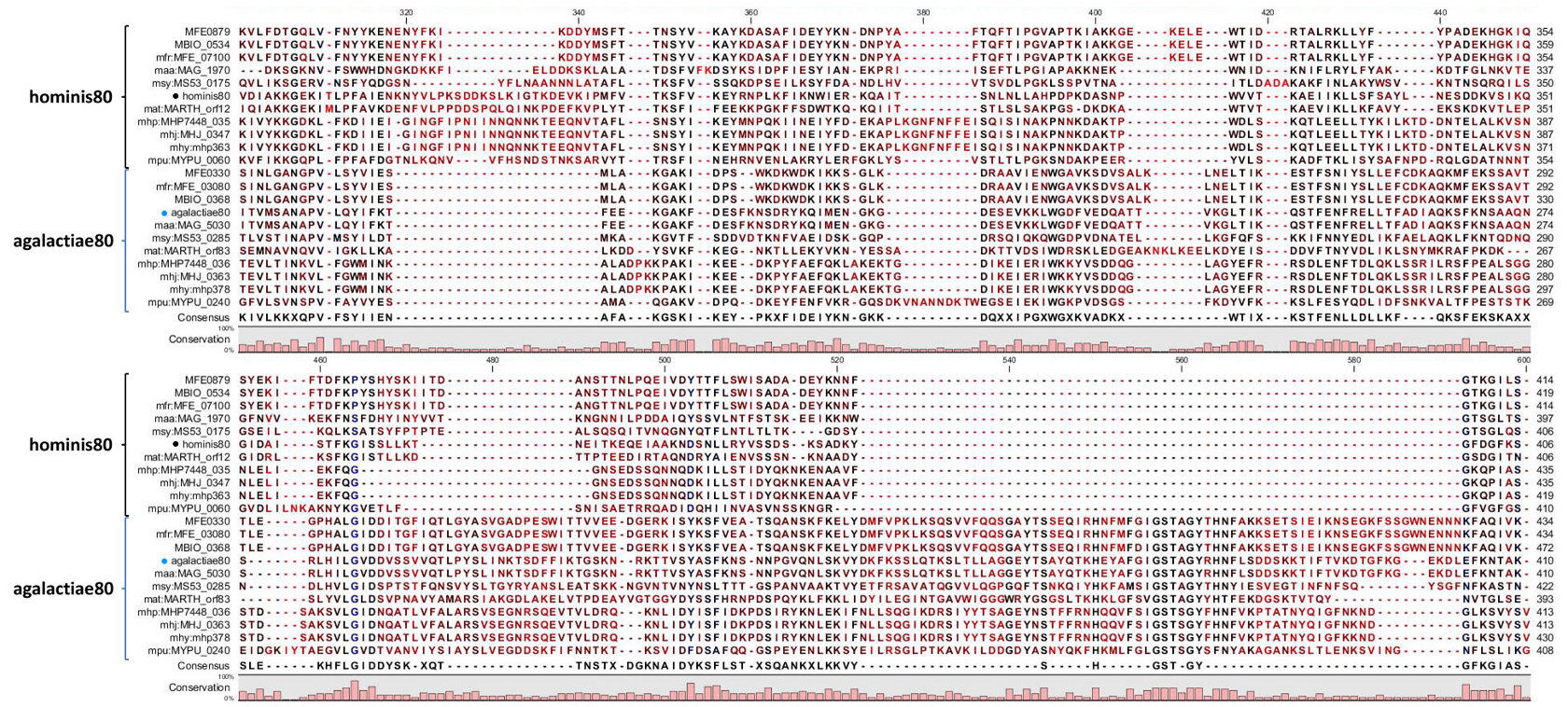

Supplement: Figure S1 — Multiple sequence alignment of the agalactiae-P80 and hominis-P80 mycoplasmal lipoproteins. The hominis-P80 cluster contains proteins similar to the M. hominis P80 and the agalactiae-P80 otherwise. Sequence conservation was shown in a gradient from blue (the most conserved), black to red (the least conserved). (TIF) [file pone.0035304.s001.tif]
