# Supplementary material for: Proteomics Characterization of Cytoplasmic and Lipid-Associated Membrane Proteins of Human Pathogen Mycoplasma fermentans M64
Source: PLoS One. 2012 Apr 20;7(4):e35304. doi: 10.1371/journal.pone.0035304 (PMC3335035; doi:10.1371/journal.pone.0035304)
Supplement: Figure S2 — COG functional profile of the 11 mycoplasmal species. The distribution of COG of total, identified and predicted-only proteins were shown as black, red and blue bars respectively. The organism names of human pathogens were labeled in red. A) COG classes related to Information Storage and Processing (J, K and L categories); B) and C) Cellular Processes (D, V, T, M, U and O categories); D) and E) Metabolism (C, G, E, F, H, I, P and Q categories) and F) Others (R, S and no COG assignment). (PDF) [file pone.0035304.s002.pdf]

Figure 2A

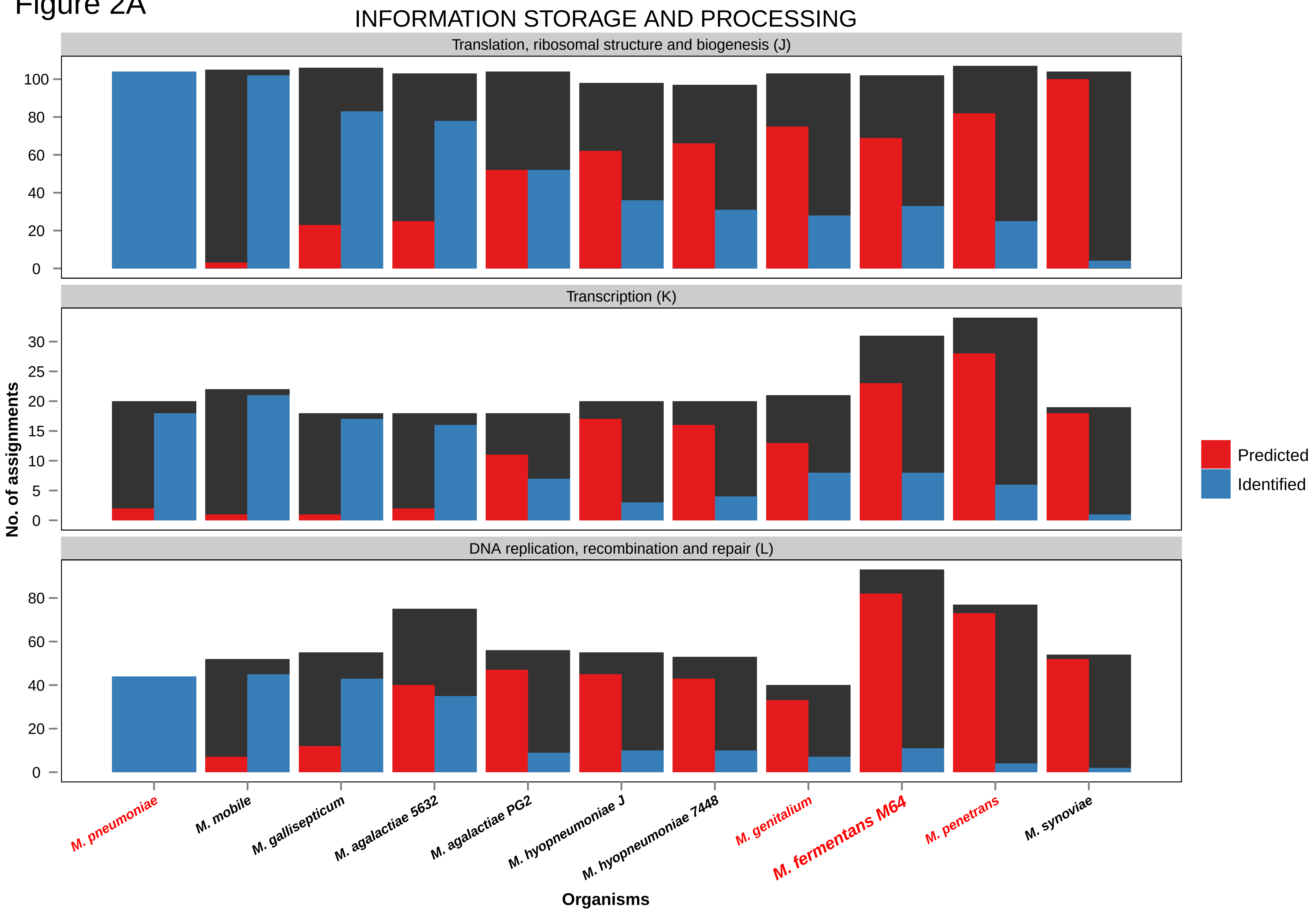
INFORMATION STORAGE AND PROCESSING
Translation, ribosomal structure and biogenesis (J)
Transcription (K)
DNA replication, recombination and repair (L)
No. of assignments
Organisms
Predicted
Identified
M. pneumoniae
M. mobile
M. gallisepticum
M. agalactiae 5632
M. agalactiae PG2
M. hyopneumoniae J
M. hyopneumoniae 7448
M. genitalium
M. fermentans M64
M. penetrans
M. synoviae

Figure 2B

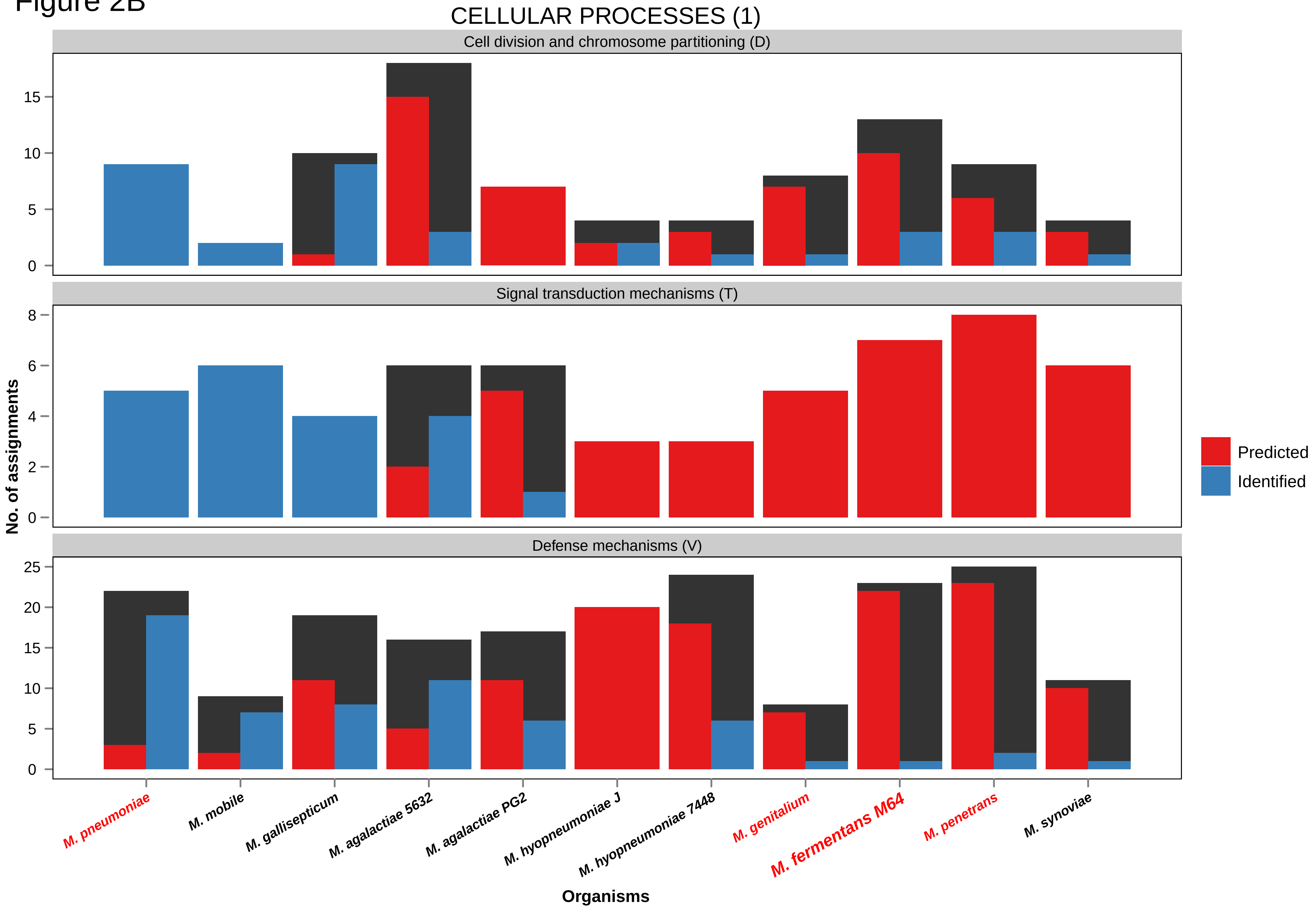

CELLULAR PROCESSES (1)
Cell division and chromosome partitioning (D)
Signal transduction mechanisms (T)
Defense mechanisms (V)
No. of assignments
0
5
10
15
0
2
4
6
8
0
5
10
15
20
25
Predicted
Identified
M. pneumoniae
M. mobile
M. gallisepticum
M. agalactiae 5632
M. agalactiae PG2
M. hyopneumoniae J
M. hyopneumoniae 7448
M. genitalium
M. fermentans M64
M. penetrans
M. synoviae
Organisms

Figure 2C

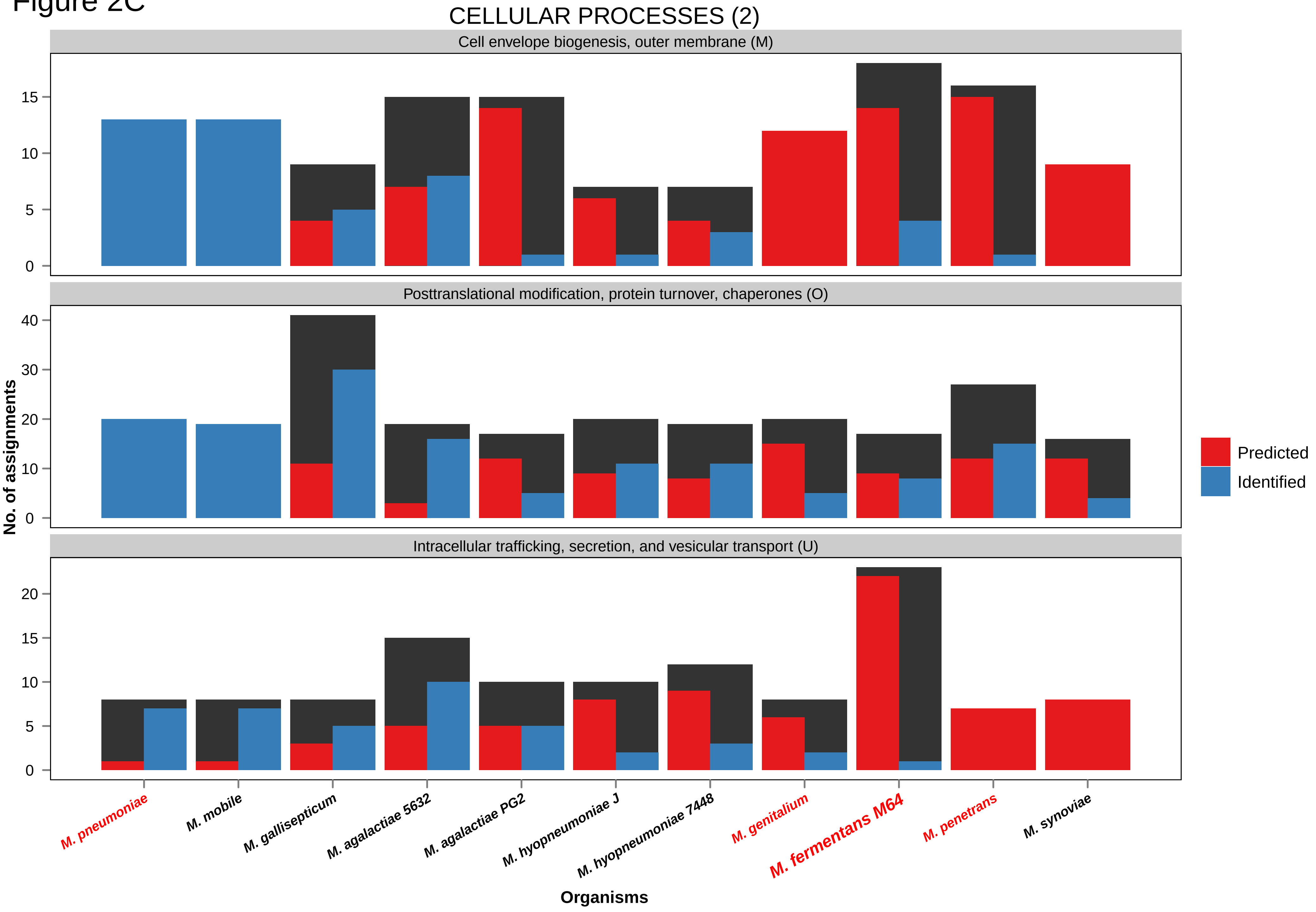

CELLULAR PROCESSES (2)
Cell envelope biogenesis, outer membrane (M)
Posttranslational modification, protein turnover, chaperones (O)
Intracellular trafficking, secretion, and vesicular transport (U)
No. of assignments
Organisms
Predicted
Identified
M. pneumoniae
M. mobile
M. gallisepticum
M. agalactiae 5632
M. agalactiae PG2
M. hyopneumoniae J
M. hyopneumoniae 7448
M. genitalium
M. fermentans M64
M. penetrans
M. synoviae

Figure 2D

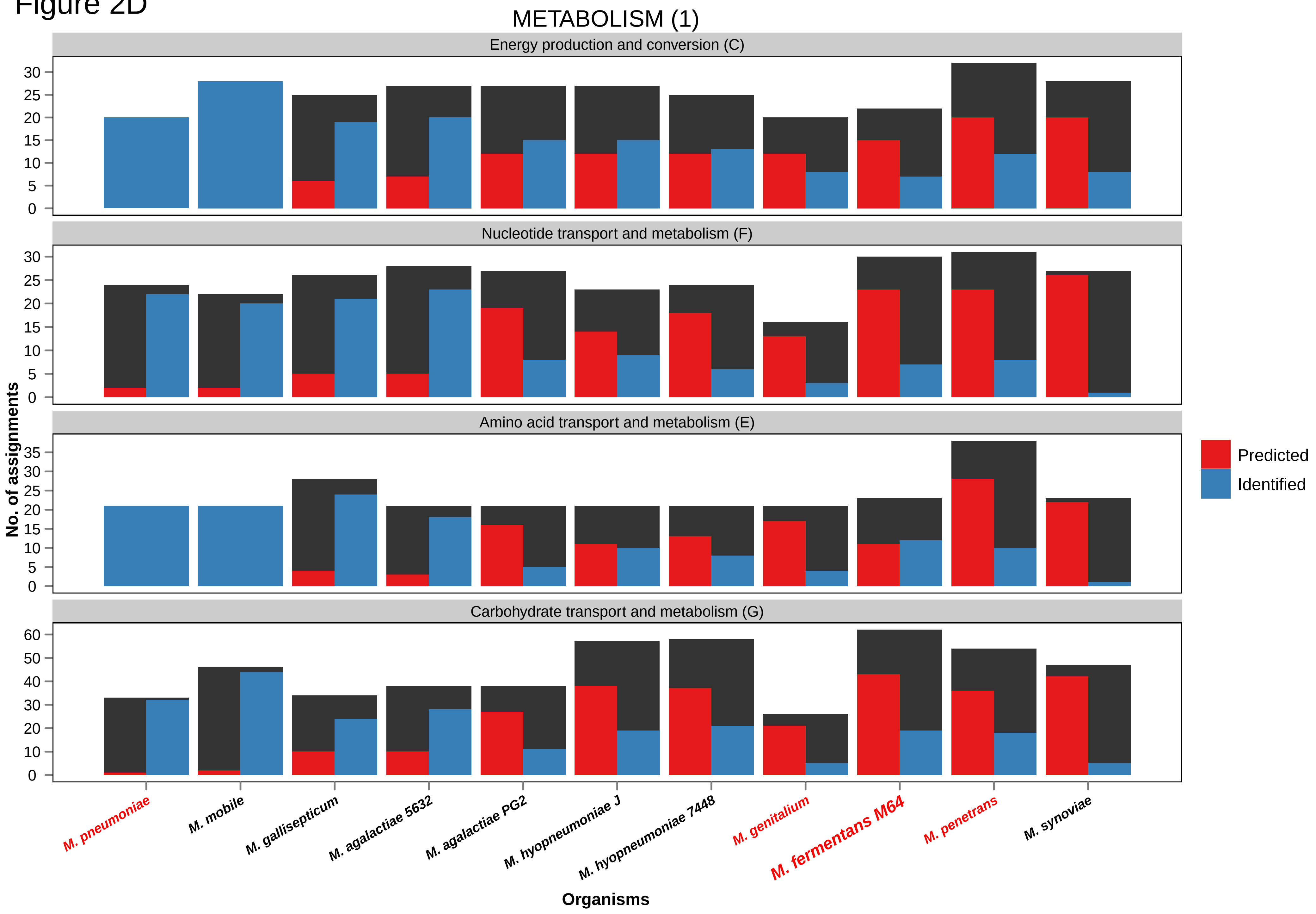

METABOLISM (1)
Energy production and conversion (C)
Nucleotide transport and metabolism (F)
Amino acid transport and metabolism (E)
Carbohydrate transport and metabolism (G)
No. of assignments
Organisms
Predicted
Identified
M. pneumoniae
M. mobile
M. gallisepticum
M. agalactiae 5632
M. agalactiae PG2
M. hyopneumoniae J
M. hyopneumoniae 7448
M. genitalium
M. fermentans M64
M. penetrans
M. synoviae

Figure 2E

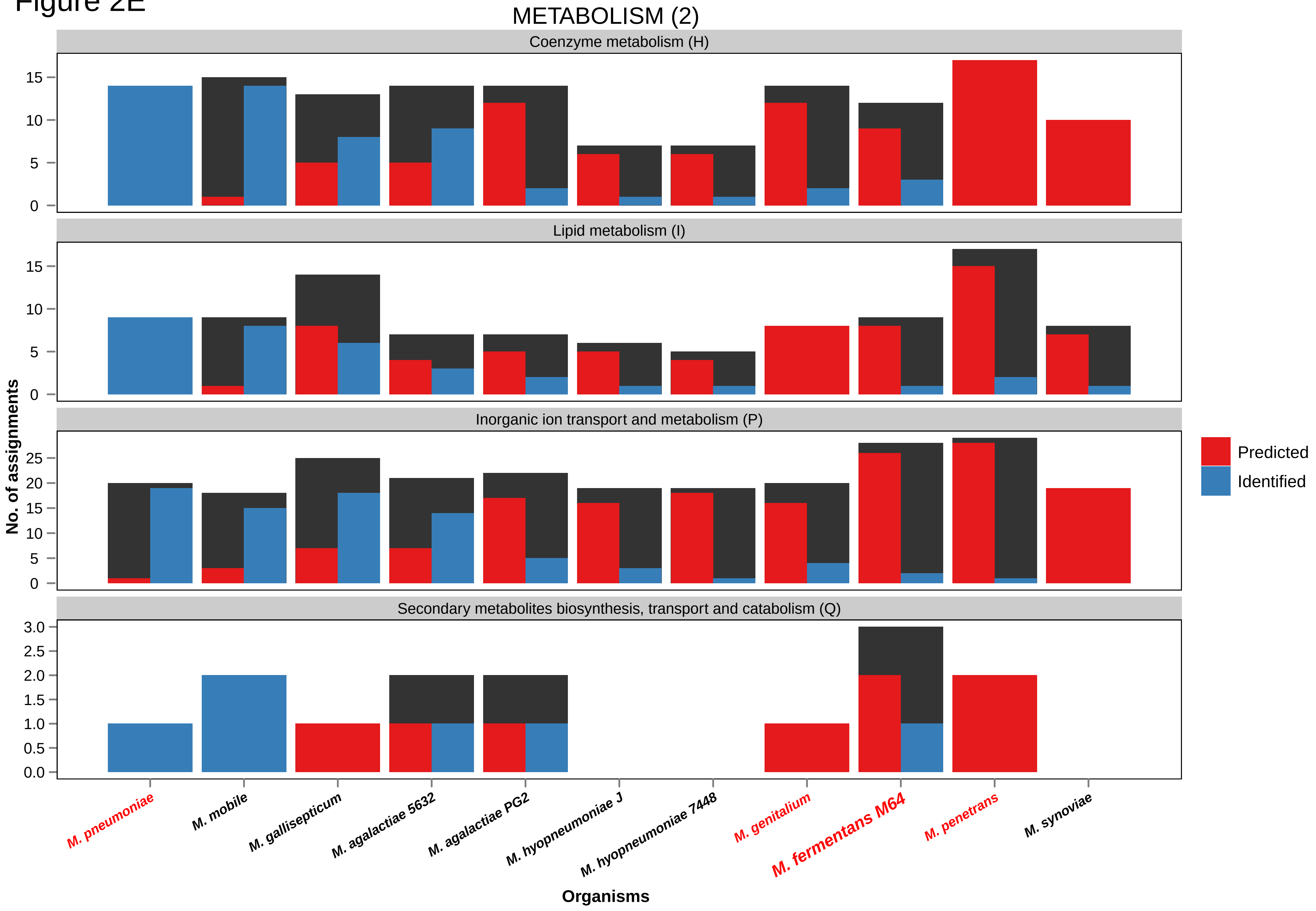

METABOLISM (2)
Coenzyme metabolism (H)
Lipid metabolism (I)
Inorganic ion transport and metabolism (P)
Secondary metabolites biosynthesis, transport and catabolism (Q)
No. of assignments
Organisms
Predicted
Identified
M. pneumoniae
M. mobile
M. gallisepticum
M. agalactiae 5632
M. agalactiae PG2
M. hyopneumoniae J
M. hyopneumoniae 7448
M. genitalium
M. fermentans M64
M. penetrans
M. synoviae

Figure 2F

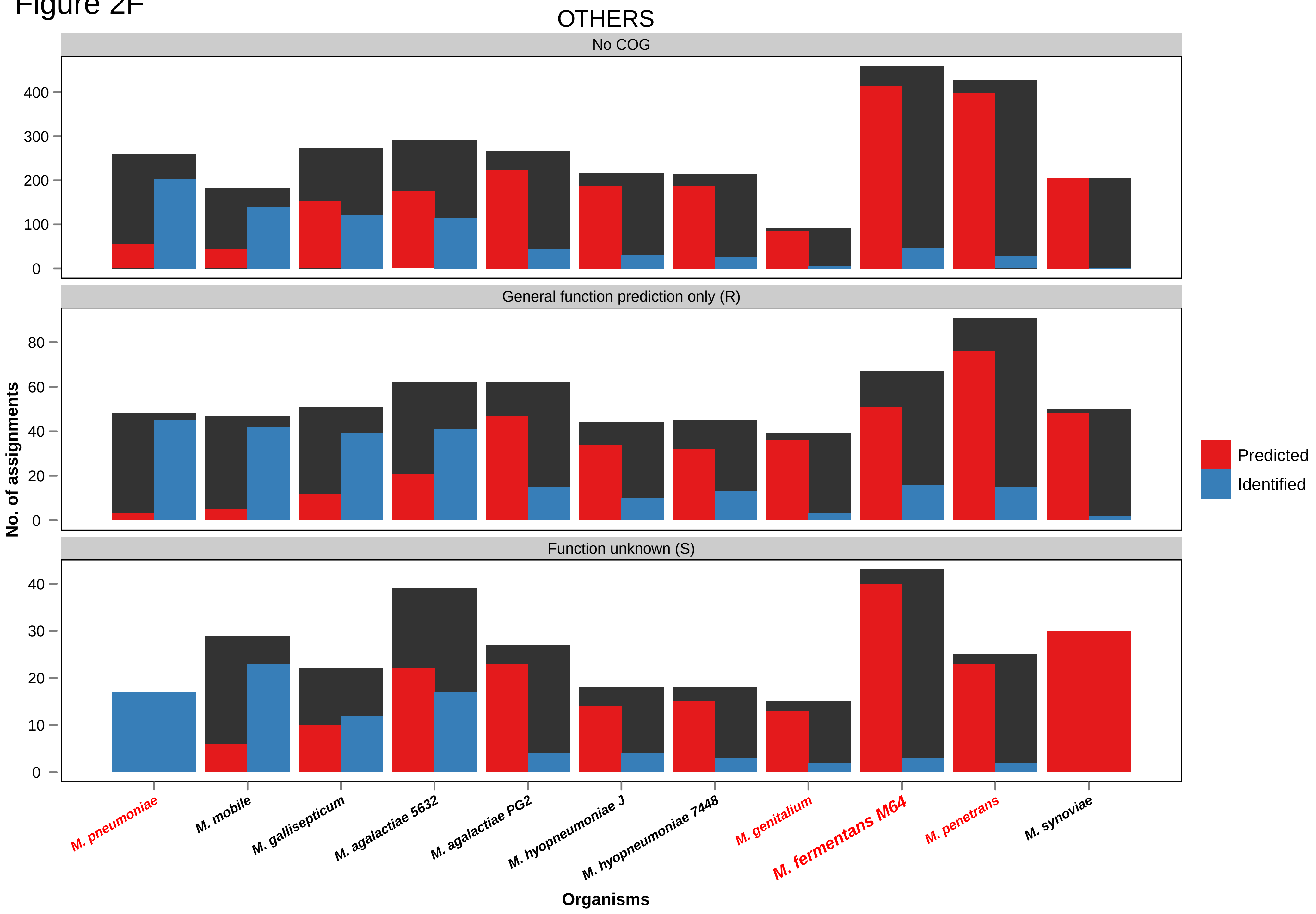

OTHERS
No COG
General function prediction only (R)
Function unknown (S)
No. of assignments
Organisms
Predicted
Identified
0
100
200
300
400
20
40
60
80
10
30
M. pneumoniae
M. mobile
M. gallisepticum
M. agalactiae 5632
M. agalactiae PG2
M. hyopneumoniae J
M. hyopneumoniae 7448
M. genitalium
M. fermentans M64
M. penetrans
M. synoviae
