# Supplementary material for: Proteomics Characterization of Cytoplasmic and Lipid-Associated Membrane Proteins of Human Pathogen Mycoplasma fermentans M64
Source: PLoS One. 2012 Apr 20;7(4):e35304. doi: 10.1371/journal.pone.0035304 (PMC3335035; doi:10.1371/journal.pone.0035304)
Supplement: Table S1 — List of Mollicutes species used in 16 S phylogenetic analysis. (DOC) [file pone.0035304.s003.doc]

**Supplementary Table 1. List of Mollicutes species used for 16S phylogenetic analysis.**

| **Organisms ^a^** | **Locus tag/GI ^b^** | **Infection/detected Host** | **PMID ^c^** |
| --- | --- | --- | --- |
| *Acholeplasma laidlawii* PG-8A | ACL_0067 | Avian | 44612 |
| Aster yellows witches'-broom phytoplasma AYWB | AYWB_r01 | Plants | 16672622 |
| *Mesoplasma florum* L1 | Mflr01 | Plants | 8863414 |
| *Mycoplasma agalactiae* 5632 | MAGa5770 | Sheep and goats | 1554763 |
| *Mycoplasma agalactiae* PG2 | MAG_r04 | Sheep and goats | 1554763 |
| *Mycoplasma alligatoris* A21JP2 | MALL_0311 | Alligators | 11321088 |
| *Mycoplasma arthritidis* 158L3-1 | MARTH_R05 | Rodents | 18573899 |
| *Mycoplasma bovis* PG45 | MBOVPG45_0956 | Cattle | 21134966 |
| *Mycoplasma capricolum* subsp. *capricolum* ATCC 27343 | MCAP_0378 | Goats, sheep and cattle | 1554763 |
| *Mycoplasma conjunctivae* HRC/581 | MCJ_007230 | Sheep and goats | 19534756 |
| *Mycoplasma crocodyli* MP145 | MCRO_0175 | Crocodile | 9226906 |
| *Mycoplasma fermentans* JER | MFE_05390 | Human | 1157581 |
| *Mycoplasma fermentans* M64* | MfeM64YM_r3 | Human | 1157581 |
| *Mycoplasma fermentans* PG18 | 238809475 | Human | 1157581 |
| *Mycoplasma gallisepticum* R | MGA_r03 | Chickens and turkeys | 44612 |
| *Mycoplasma genitalium* G37* | MG_rrnA16S | Human | 2687748 |
| *Mycoplasma haemofelis* Langford 1 | HF1_r02 | Cat | 21317334 |
| *Mycoplasma hominis* ATCC 23114 | MHO_r02 | Human | 1157581 |
| *Mycoplasma hyopneumoniae* 232 | mhprRNA-16S | Pigs | 15489423 |
| *Mycoplasma hyopneumoniae* 7448 | MHP7448_r3 | Pigs | 16077101 |
| *Mycoplasma hyopneumoniae* J | MHJ_0709 | Pigs | 16077101 |
| *Mycoplasma hyorhinis* HUB-1 | MHR_r001 | Pigs | 20802032 |
| *Mycoplasma leachii* PG50 | MSB_A0392 | Cattle | 19502315 |
| *Mycoplasma mobile* 163K | MMOB9320 | Fish | 6502137 |
| *Mycoplasma mycoides* subsp. *capri* LC 95010 | MLC_0626 | Goats | 21324191 |
| *Mycoplasma mycoides* subsp. *mycoides* SC str. PG1 | MSC_rrnA-16S | Cattle | 1554763 |
| *Mycoplasma penetrans* HF-2* | MYPE20020 | Human | 12466555 |
| *Mycoplasma pneumoniae* M129* | MPNr01 | Human | 1157581 |
| *Mycoplasma pulmonis* UAB CTIP | MYPU_RRNA_16S | Rodents | 11353084 |
| *Mycoplasma suis* Illinois | MSU_0572 | Pigs | 21317328 |
| *Mycoplasma suis* KI3806 | Msuir01 | Pigs | 21398558 |
| *Mycoplasma synoviae* 53 | MS53_0724 | Chickens and turkeys | 44612 |
| Onion yellows phytoplasma OY-M | PAM_r01 | Plants | 14661021 |
| *Spiroplasma citri* Tioman | 313510861 | Madagascar periwinkle | 18944838 |
| *Spiroplasma kunkelii* CR2-3x | 83939664 | Maize | 12676553 |
| *Ureaplasma parvum* serovar 3 ATCC 27815 | UPA3_0117 | Human | 11931172 |
| *Ureaplasma parvum* serovar 3 ATCC 700970 | UUr01 | Human | 11048724 |
| *Ureaplasma urealyticum* serovar 10 ATCC 33699 | UUR10_0124 | Human | 11931172 |

1. Human-host mycoplasmas with published proteome data were labeled with asterisk (*).
2. The 16S ribosomal gene sequence IDs that were used in the phylogenetic analysis.
3. A PMID (PubMed identifier) is a unique number assigned to each PubMed citation of life sciences and biomedical journal articles
