# Supplementary material for: Proteomics Characterization of Cytoplasmic and Lipid-Associated Membrane Proteins of Human Pathogen Mycoplasma fermentans M64
Source: PLoS One. 2012 Apr 20;7(4):e35304. doi: 10.1371/journal.pone.0035304 (PMC3335035; doi:10.1371/journal.pone.0035304)
Supplement: Table S3 — Conservation of M. fermentans M64 proteins conserved across the fully sequenced Mycoplasma species. The values showed the number and percentage of identified and predicted M. fermentans M64 proteins that are homologous with species in each row. The two percentage columns were colored according to the level of conservation, ranging from dark red (highest conservation) to dark green (lowest conservation). (DOC) [file pone.0035304.s005.doc]

**Supplementary Table 3. Conservation of *M. fermentans* M64 proteins across the fully sequenced mycoplasmal species. The values showed the number and percentage of identified and predicted *M. fermentans* M64 proteins that are homologous with species in each row. The two percentage columns were colored according to the level of conservation, ranging from dark red (highest conservation) to dark green (lowest conservation).**

| **Code** | **Organisms ^a^** | **Identified Proteins ^b^** | **% Identified Proteins** | **Predicted Proteins ^c^** | **% Predicted Proteins** |
| --- | --- | --- | --- | --- | --- |
| mal | *Mycoplasma agalactiae* 5632 | 121 | 66.85% | 476 | 45.33% |
| maa | *Mycoplasma agalactiae* PG2 | 120 | 66.30% | 439 | 41.81% |
| mat | *Mycoplasma arthritidis* | 102 | 56.35% | 323 | 30.76% |
| mbv | *Mycoplasma bovis* PG45 | 118 | 65.19% | 470 | 44.76% |
| mcp | *Mycoplasma capricolum* | 91 | 50.28% | 268 | 25.52% |
| mco | *Mycoplasma conjunctivae* | 101 | 55.80% | 314 | 29.90% |
| mcd | *Mycoplasma crocodyli* | 118 | 65.19% | 430 | 40.95% |
| mfr | ***Mycoplasma fermentans* JER** | 162 | 89.50% | 756 | 72.00% |
| mbi | ***Mycoplasma fermentans* PG18** | 178 | 98.34% | 898 | 85.52% |
| mga | *Mycoplasma gallisepticum* | 85 | 46.96% | 234 | 22.29% |
| mge | ***Mycoplasma genitalium*** | 85 | 46.96% | 209 | 19.90% |
| mha | *Mycoplasma haemofelis* | 57 | 31.49% | 131 | 12.48% |
| mho | ***Mycoplasma hominis*** | 103 | 56.91% | 315 | 30.00% |
| mhy | *Mycoplasma hyopneumoniae* 232 | 100 | 55.25% | 293 | 27.90% |
| mhp | *Mycoplasma hyopneumoniae* 7448 | 99 | 54.70% | 298 | 28.38% |
| mhj | *Mycoplasma hyopneumoniae* J | 100 | 55.25% | 298 | 28.38% |
| mhr | *Mycoplasma hyorhinis* | 107 | 59.12% | 328 | 31.24% |
| mlc | *Mycoplasma leachii* | 91 | 50.28% | 266 | 25.33% |
| mmo | *Mycoplasma mobile* | 97 | 53.59% | 332 | 31.62% |
| mmy | *Mycoplasma mycoides* subsp. *mycoides* SC PG1 | 88 | 48.62% | 257 | 24.48% |
| mpe | ***Mycoplasma penetrans*** | 89 | 49.17% | 277 | 26.38% |
| mpn | ***Mycoplasma pneumoniae*** | 83 | 45.86% | 215 | 20.48% |
| mpu | *Mycoplasma pulmonis* | 102 | 56.35% | 358 | 34.10% |
| mss | *Mycoplasma suis* Illinois | 50 | 27.62% | 106 | 10.10% |
| msk | *Mycoplasma suis* KI3806 | 49 | 27.07% | 100 | 9.52% |
| msy | *Mycoplasma synoviae* | 105 | 58.01% | 366 | 34.86% |

1. Human pathogens are highlighted in bold font
2. The 181 identified *M. fermentans* M64 proteins were used to BLASTP against the other Mycoplasmal predicted proteome to identify homologs in each genome
3. The 1050 predicted *M. fermentans* M64 proteins were used to BLASTP against the other Mycoplasmal predicted proteome to identify homologs in each genome
