# Supplementary material for: Proteomics Characterization of Cytoplasmic and Lipid-Associated Membrane Proteins of Human Pathogen Mycoplasma fermentans M64
Source: PLoS One. 2012 Apr 20;7(4):e35304. doi: 10.1371/journal.pone.0035304 (PMC3335035; doi:10.1371/journal.pone.0035304)
Supplement: Table S5 — List of enzymes that participate in the nucleoside salvage pathway. (DOC) [file pone.0035304.s007.doc]

**Supplementary Table 5**. List of enzymes that participate in the nucleoside salvage pathway.

| **No. ^a)^** | **Gene** | **Description** | **ORF ^b)^** |
| --- | --- | --- | --- |
| 1 | *deoA*  *deoD* | Pyrimidine-nucleoside phosphorylase  Purine-nucleoside phosphorylase | MfeM64YM0502*  MfeM64YM0503*, MfeM64YM0968* |
| 2 | *apt*  *upp hpt* | Adenine phosphoribosyltransferase  Uracil phosphoribosyltransferase  Hypoxanthine phosphoribosyltransferase | MfeM64YM0075  MfeM64YM0403  MfeM64YM0509* |
| 3 | *adk*  *dak*  *gsk*  *dgk*  *tdk*  *udk*  *dck* | Adenosine kinase  Deoxyadenosine kinase  Inosine-guanosine kinase  Deoxyguanosine kinase  Thymidine kinase  Uridine-cytidine kinase  Deoxycytidine kinase | NF  NF  NF  MfeM64YM0510, MfeM64YM0511*  MfeM64YM0921*  NF  NF |
| 4 | *ushA* | 5'-nucleotidase | NF |
| 5 | *cmk*  *adk*  *pyrH*  *gmk*  *tmk* | Cytidylate kinase  Adenylate kinase  Uridylate kinase  Guanylate kinase  Thymidylate kinase | MfeM64YM0167*  MfeM64YM0741*  MfeM64YM0877*  MfeM64YM0903  MfeM64YM0974* |
| 6 | *ndk*  *pyk* | Nucleoside-diphosphate kinase  Pyruvate kinase | NF  MfeM64YM0189* |
| 7 | *nrd* | Ribonucleotide reductase (RNR) | NF |

1. Numbers correspond to the enzyme labeling used in Figure 5.
2. Proteins Identified in this study were marked with asterisk (*). Enzymes not found in predicted ORFs of *M. fermentans* M64 was labeled NF (not found).
