# Supplementary material for: Proteomics Characterization of Cytoplasmic and Lipid-Associated Membrane Proteins of Human Pathogen Mycoplasma fermentans M64
Source: PLoS One. 2012 Apr 20;7(4):e35304. doi: 10.1371/journal.pone.0035304 (PMC3335035; doi:10.1371/journal.pone.0035304)
Supplement: Table S6 — List of enzymes that participate in the glycolysis pathway. (DOC) [file pone.0035304.s008.doc]

**Supplementary Table 6**. List of enzymes that participate in the glycolysis pathway.

| **No. ^a)^** | **Gene** | **Description** | **ORF ^b)^** |
| --- | --- | --- | --- |
| 1 | *crr*  *ptsG* | PTS system, glucose-specific IIABC component | MfeM64YM0040, MfeM64YM0278*, MfeM64YM0539, MfeM64YM0893 |
| 2 | *pgi* | Glucose-6-phosphate isomerase | MfeM64YM0420* |
| 3 | *pfkA* | 6-phosphofructokinase | MfeM64YM0504 |
| 4 | *fba* | Fructose-bisphosphate aldolase | MfeM64YM0187* |
| 5 | *tpiA* | Triosephosphate isomerase | MfeM64YM0488* |
| 6 | *gap* | Glyceraldehyde-3-phosphate dehydrogenase | MfeM64YM0896* |
| 7 | *pgk* | Phosphoglycerate kinase | MfeM64YM0492* |
| 8 | *gpmI* | 2,3-bisphosphoglycerate-independent phosphoglycerate mutase | MfeM64YM0293* |
| 9 | *eno* | Enolase | MfeM64YM0252* |
| 10 | *pyk* | Pyruvate kinase | MfeM64YM0189* |
| 11 | *pdhA*  *pdhB* | Pyruvate dehydrogenase E1-alpha subunit  Pyruvate dehydrogenase E1-beta subunit | MfeM64YM0830*  MfeM64YM0829* |
| 12 | *pdhC* | Dihydrolipoamide acetyltransferase | MfeM64YM0828 |
| 13 | *pdhD* | Dihydrolipoamide dehydrogenase | MfeM64YM0827 |
| 14 | *glck* | Glucokinase | MfeM64YM1012 |

1. Numbers correspond to the enzyme labeling used in Figure 6.
2. Identified proteins were marked with asterisk (*).
