# Supplementary material for: Proteomics Characterization of Cytoplasmic and Lipid-Associated Membrane Proteins of Human Pathogen Mycoplasma fermentans M64
Source: PLoS One. 2012 Apr 20;7(4):e35304. doi: 10.1371/journal.pone.0035304 (PMC3335035; doi:10.1371/journal.pone.0035304)
Supplement: Table S7 — List of gene locus IDs that were used in the agalactiae-P80 and hominis-P80 lipoprotein multiple sequence alignment. (DOC) [file pone.0035304.s009.doc]

**Supplementary Table 7. List of genes that are homologous to the *M. hominis* *hitABL* operon and *M. agalactiae* *ma-mp81* gene**.

| **Organisms** | **KEGG Abbreviation** | **hominis-P80 (GI: CAB92784)** | ***hitB* (P60)** | ***hitL* (HinT)** | **agalactiae-P80 (GI: CAC38110)** |
| --- | --- | --- | --- | --- | --- |
| *Mycoplasma agalactiae* 5632 | mal | MAGa1970 | MAGa1980 | MAGa1990 | MAGa5500 |
| *Mycoplasma agalactiae* PG2 | maa | MAG_1970 ^#^ | MAG_1980 | MAG_1990 | MAG_5030 ^#^ |
| *Mycoplasma arthritidis* | mat | MARTH_orf123 ^#^ | MARTH_orf125 | MARTH_orf126 | MARTH_orf832 ^#^ |
| *Mycoplasma bovis* Hubei-1 | mbh | MMB_0202 | MMB_0203 | MMB_0204 | MMB_0540 |
| *Mycoplasma bovis* PG45 | mbv | MBOVPG45_0646 | MBOVPG45_0645 | MBOVPG45_0644 | MBOVPG45_0311 |
| *Mycoplasma capricolum* | mcp | MCAP_0470 | MCAP_0471 | MCAP_0472 | MCAP_0451 |
| *Mycoplasma conjunctivae* | mco | MCJ_005940 | MCJ_005930 | MCJ_005920 | MCJ_000120 MCJ_000140 |
| *Mycoplasma crocodyli* | mcd | MCRO_0746 | MCRO_0745 | MCRO_0744 | MCRO_0707 |
| *Mycoplasma fermentans* JER | mfr | MFE_07100 ^#^ | MFE_07110 | MFE_07120 | MFE_03080 ^#^ |
| *Mycoplasma fermentans* M64 | mfm | MfeM64YM_0879 ^#^ | MfeM64YM_0880 | MfeM64YM_0881 | MfeM64YM_0330 ^#^ |
| *Mycoplasma fermentans* PG18 | NA | MBIO_0534 ^#^ | MBIO_0533 | MBIO_0532 | MBIO_0368 ^#^ |
| *Mycoplasma gallisepticum* | mga | NA | NA | MGA_0212 | MGA_0674 |
| *Mycoplasma genitalium* | mge | MG_133 * | | MG_132 | MG_260 |
| *Mycoplasma haemofelis* | mha | HF1_11030 * | | HF1_11020 | NA |
| *Mycoplasma hominis* | mho | MHO_3500 | MHO_3490 | MHO_3480 | MHO_0720 |
| *Mycoplasma hyopneumoniae* 232 | mhy | mhp363 ^#^ | mhp364 | mhp365 | mhp378 ^#^ |
| *Mycoplasma hyopneumoniae* 7448 | mhp | MHP7448_0352 ^#^ | MHP7448_0353 | MHP7448_0354 | MHP7448_0367 ^#^ |
| *Mycoplasma hyopneumoniae* J | mhj | MHJ_0347 ^#^ | MHJ_0348 | MHJ_0349 | MHJ_0363 ^#^ |
| *Mycoplasma hyorhinis* | mhr | MHR_0628 | MHR_0627 | MHR_0626 | MHR_0062 |
| *Mycoplasma leachii* | mlc | MSB_A0483 | MSB_A0484 | MSB_A0485 | MSB_A0484 |
| *Mycoplasma mobile* | mmo | MMOB0910 | MMOB0900 | MMOB0890 | NA |
| *Mycoplasma mycoides* subsp. *capri* LC 95010 | mml | MLC_4800 | MLC_4790 | MLC_4780 | MLC_4970 |
| *Mycoplasma mycoides* subsp. *mycoides* SC PG1 | mmy | MSC_0500 | MSC_0499 | MSC_0498 | MSC_0519 |
| *Mycoplasma pneumoniae* | mpn | MPN274 * | | MPN273 | MPN200 MPN284 |
| *Mycoplasma pulmonis* | mpu | MYPU_0060 ^#^ | MYPU_0070 | MYPU_0080 | MYPU_0240 ^#^ |
| *Mycoplasma suis* Illinois | mss | NA | NA | MSU_0468 | NA |
| *Mycoplasma suis* KI3806 | msk | NA | NA | Msui04070 | NA |
| *Mycoplasma synoviae* | msy | MS53_0175 ^#^ | MS53_0174 | MS53_0173 | MS53_0285 ^#^ MS53_0329 |

* Gene that encodes an integral pore-forming protein and does not have sequence similarity with *hitA* and/or *hitB*.

^#^ Gene whose deduced amino acid sequence was used in the multiple sequence alignment.
