# Supplementary material for: Proteomics Characterization of Cytoplasmic and Lipid-Associated Membrane Proteins of Human Pathogen Mycoplasma fermentans M64
Source: PLoS One. 2012 Apr 20;7(4):e35304. doi: 10.1371/journal.pone.0035304 (PMC3335035; doi:10.1371/journal.pone.0035304)
Supplement: Table S8 — Predicted protein antigenicity and continuous B-cell epitopes of identified lipoproteins from Mycoplasma fermentans M64. The data was sorted by the predicted antigenicity likelihood of the lipoproteins. (DOC) [file pone.0035304.s010.doc]

**Supplementary Table 8. Predicted protein antigenicity and continuous B-cell epitopes of identified lipoproteins from *Mycoplasma fermentans* M64. The data was sorted by the predicted antigenicity likelihood of the lipoproteins.**

| **ORF ID ^a)^** | **Length** | **Top B-cell epitopes** | | **Second B-cell epitopes** | | **Third B-cell epitopes** | | **Antigenicity Likelihood** |
| --- | --- | --- | --- | --- | --- | --- | --- | --- |
|  |  | **Location ^b)^** | **Pattern** | **Location ^b)^** | **Pattern** | **Location ^b)^** | **Pattern** |  |
| MfeM64YM0330 | 731 | 711 | GQYKDQ | 712 | QYKDQI | 96 | PGFKEV | 0.945 |
| MfeM64YM0281 | 929 | 204 | GNETKY | 204 | GNETKYD | 462 | NQQQQLD | 0.914 |
| MfeM64YM0451 | 522 | 471 | DKSFKE | 152 | LNPEYKD | 230 | TSKTFKD | 0.894 |
| MfeM64YM0802 | 522 | 471 | DKSFKE | 152 | LNPEYKD | 230 | TSKTFKD | 0.894 |
| MfeM64YM0846 | 522 | 471 | DKSFKE | 152 | LNPEYKD | 230 | TSKTFKD | 0.894 |
| MfeM64YM0380 | 522 | 471 | DKSFKE | 152 | LNPEYKD | 230 | TSKTFKD | 0.883 |
| MfeM64YM0714 | 791 | 414 | KVDQDS | 548 | KLNEAN | 1 | SNFKKT | 0.881 |
| MfeM64YM0331 | 392 | 258 | PAGTKV | 232 | AVGKEI | 231 | KAVGKEI | 0.872 |
| MfeM64YM0433 | 392 | 152 | QAQYEN | 337 | NSLSKN | 337 | NSLSKNV | 0.868 |
| MfeM64YM0300 | 891 | 91 | NLKADK | 227 | GFKKET | 90 | DNLKADK | 0.841 |
| MfeM64YM1013 | 214 | 44 | GKDYNF | 44 | GKDYNFS | 69 | NNPEFKK | 0.789 |
| MfeM64YM0978 | 428 | 386 | KELPEDF | 387 | ELPEDF | 351 | GTQKEK | 0.761 |
| MfeM64YM0039 | 241 | 63 | KIKNEV | 48 | NKNYAGE | 71 | GFDKIT | 0.743 |
| MfeM64YM0336 | 498 | 368 | GVNEET | 80 | PHRAKV | 142 | QHPNEVG | 0.646 |
| MfeM64YM0984 | 680 | 188 | EYDAEK | 565 | GFSEKS | 188 | EYDAEKA | 0.586 |
| MfeM64YM0616 | 591 | 240 | SLDAKI | 240 | SLDAKIK | 241 | LDAKIKD | 0.540 |
| MfeM64YM0021 | 244 | 109 | QDKYKT | 57 | TVTDET | 57 | TVTDETK | 0.510 |
| MfeM64YM0871 | 665 | 40 | DYDKKE | 377 | PDSFKI | 390 | SDFSTL | 0.488 |
| MfeM64YM0688 | 289 | 52 | EISEKE | 200 | PEIKSK | 200 | PEIKSKV | 0.444 |
| MfeM64YM0895 | 233 | 119 | EDGIAS | 83 | PSDVDN | 45 | ADASKYS | 0.375 |
| MfeM64YM0519 | 127 | 65 | KITDSA | 84 | KMKSEN | 85 | MKSENY | 0.275 |

1. ORF IDs are listed in the order of their predicted antigenicity likelihood.
2. Numbers denote the starting amino acid position of the predicted B-cell epitope pattern in the peptide.
